# Unleashing the industrial potential of microbial plant cell wall polysaccharide-degrading enzymes: recent advances in protein engineering

**DOI:** 10.3389/fmicb.2026.1945309

**Published:** 2026-10-13

**Authors:** Zihao Huang, Liangtao Xu, Jun Zhou, Renjun Gao, Huaibin Shi

**Affiliations:** 1 China Tobacco Jiangsu Industrial Co., Ltd., Nanjing, China; 2 State Key Laboratory of Analytical Chemistry for Life Science, School of Chemistry, Nanjing University, Nanjing, China; 3 Key Laboratory for Molecular Enzymology and Engineering of Ministry of Education, School of Life Sciences, Jilin University, Changchun, China

**Keywords:** directed evolution, enzyme fusion, industrial feasibility, microbial plant cell wall polysaccharide-degrading enzymes, rational design, semi-rational design

## Abstract

Plant cell wall polysaccharide-degrading enzymes (PCWPEs) used in industry are predominantly of microbial origin. Key enzyme classes, including cellulases, lytic polysaccharide monooxygenases (LPMOs), β-xylanases, β-mannanases and pectinases, have attained technological maturity and are widely deployed across agro-waste biorefineries, food and feed processing, textile and pulp manufacturing. Low catalytic efficiency, insufficient thermostability, poor operational adaptability, restricted functionality, and product inhibition limit the industrial use of these enzymes. Structure- and sequence-guided design, supported by computational design tools, now allows more targeted improvement of PCWPE performance. Yet, the stringent and context-specific performance requirements imposed by diverse industrial applications continue to present substantial challenges. This review systematically summarizes current engineering strategies for PCWPEs in the past decade, aiming to provide a comprehensive understanding of the tailored strategies employed in different engineering directions and to offer insightful proposals for future research.

## Graphical Abstract

**Figure:**
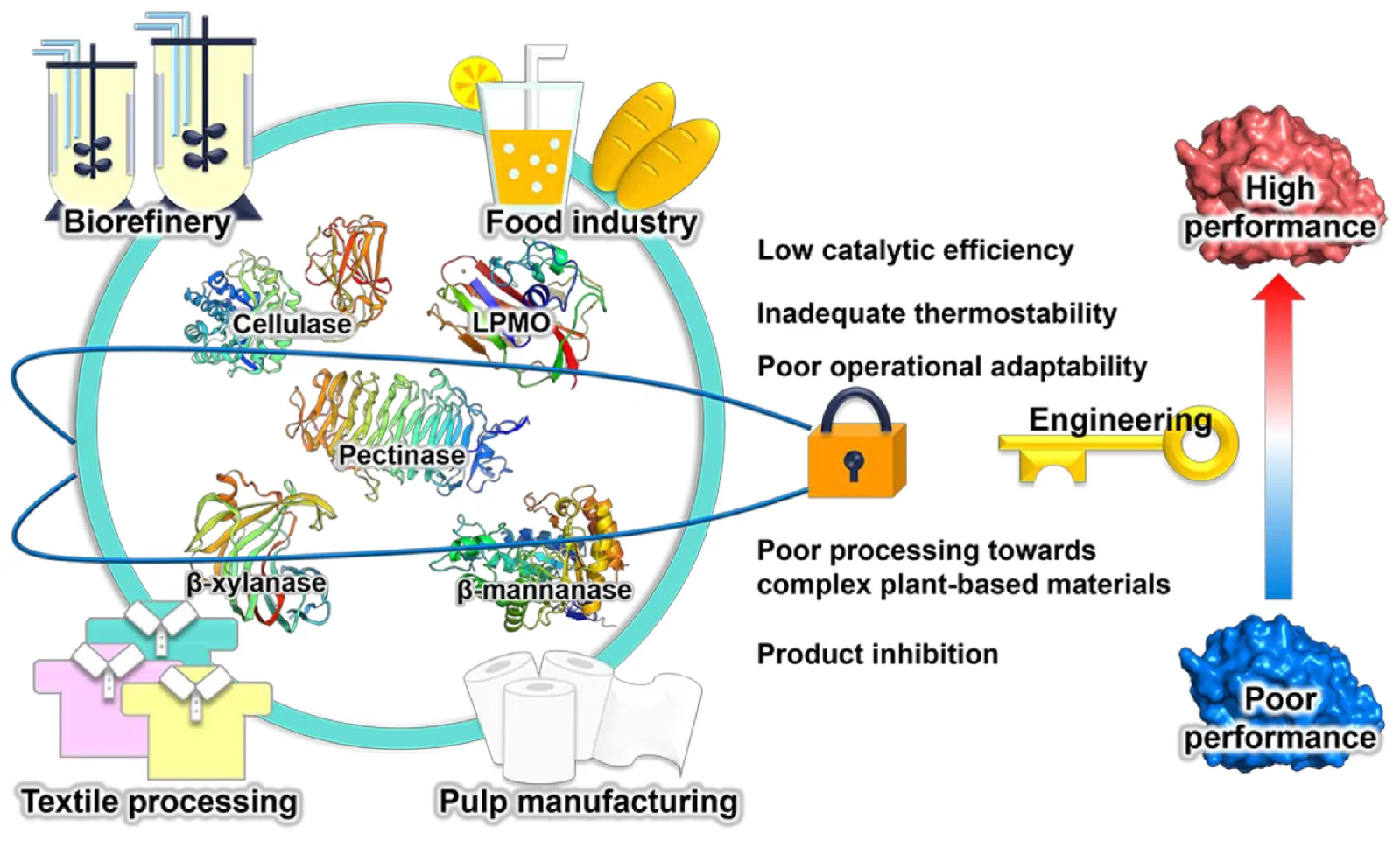
Protein engineering represents a promising strategy to overcome the industrial limitations associated with PCWPEs.

## 1 Introduction

Plant cell wall polysaccharides are mainly divided into cellulose, hemicellulose, and pectin. Cellulose, the primary structural component, forms microfibrils (Robert et al., 2004) that intertwine with hemicellulose and create a strong network for mechanical support of plant cells (Scheller and Ulvskov, 2010). Heterogeneous pectins play pivotal roles in regulating cell adhesion, porosity, and hydration (Willats et al., 2001). Plant cell wall polysaccharide-degrading enzymes (PCWPEs) catalyze the degradation of corresponding polysaccharides, thereby enhancing the industrial availability of plant-based materials for different industrial processes (Harris and Smith, 2006; Somerville et al., 2010; Kumar et al., 2016). Cellulases, hemicellulases and pectinases, primarily mediate the degradation of their cognate glycans (Kikot et al., 2009). In contrast, LPMOs enable oxidative cleavage of glycosidic bonds within diverse cell wall matrix glycans, expanding the catalytic capacity beyond classical hydrolases or lyases (Fanuel et al., 2017). Currently, microbial PCWPEs serve as the predominant biocatalysts across diverse industrial sectors. This predominance stems from their advantageous properties including controllable production costs (Bischof et al., 2016), functionally comprehensive enzyme systems (Nargotra et al., 2023), rich diversity (Levasseur et al., 2013), and well-established expression platforms (Lambertz et al., 2014). Future market expansion will be driven by increasing demand for green, sustainable processes like efficient agro-waste valorization, pulp bleaching, and high-quality food processing (Samanta, 2019; Srivastava et al., 2023; Mu et al., 2024; Mahima et al., 2026). PCWPE research has concentrated mainly on cellulases, lytic polysaccharide monooxygenases (LPMOs), β-xylanases, β-mannanases, and pectinases that have achieved a high degree of industrial maturity in terms of process robustness, catalytic efficiency, and cost-effectiveness. In the context of hemicellulose-degrading enzymes, core endo-acting enzymes targeting the backbone (e.g., β-xylanases and β-mannanases) have been prioritized for their direct and quantifiable impact on sugar yields. In contrast, auxiliary enzymes, including β-xylosidases, α-L-arabinofuranosidases, acetyl xylan esterases, and feruloyl esterases, enable full hemicellulose deconstruction but exert less direct, measurable effects on yield. Though historically underemphasized relative to core backbone-cleaving enzymes, their essential synergistic role in maximizing saccharification efficiency is now increasingly recognized. Accordingly, this review focuses on the enzyme families that have already been empirically verified in relevant industrial fields and possess clear application potential.

The inherent limitations of native microbial PCWPEs pose significant constraints on their industrial deployment. Among them, suboptimal catalytic efficiency represents a key bottleneck, particularly when benchmarked against conventional acid hydrolysis (Hoang et al., 2021), directly compromising the effectiveness of biomass conversion. Additionally, most native PCWPEs exhibit inadequate thermostability under the elevated temperatures routinely employed in industrial bioprocessing (Che Hussian and Leong, 2023). Some PCWPEs also have narrow substrate spectra or limited functions, thereby reducing their ability to process plant materials with complex compositions and structures (Imran et al., 2019). In specific scenarios, the limited adaptability of many PCWPEs to specific temperature and pH conditions (Giovannoni et al., 2020), along with their susceptibility to salts (Domínguez and de María, 2013) and organic solvents (Vaidya et al., 2022), often compromises their enzymatic activity under operational conditions. Product accumulation during prolonged processes such as biorefining can reduce the activity of some PCWPEs and limit treatment efficiency (Atreya et al., 2016).

Enzyme engineering enables the precise modification of enzymatic properties at the molecular level to meet stringent industrial requirements (Ali et al., 2020). Directed evolution, a well-established and empirically validated methodology, employs multiple rounds of random mutagenesis and high-throughput screening to find out the best candidate used under harsh industrial conditions (Arnold, 2018). Rational design uses structural information and computational design tools to introduce targeted changes that optimize selected properties (Song et al., 2023; Vecchietti et al., 2024). Enzyme fusion combines multiple enzyme-coding sequences to produce multifunctional chimeras for complex reactions (Ma et al., 2022). Engineering of microbial PCWPEs has attracted increasing scholarly attention over the past decade, as evidenced by the steady growth in publication output and citation counts (Figure 1). However, compared to traditional and well-characterized industrial enzymes such as lipase, protease, and amylase, the development and application of PCWPEs remain more complex. This complexity arises not only from their intricate molecular structures but also from the heterogeneous nature of cell wall polysaccharides (Burton et al., 2010). Over the past decade, growing attention to green, low-carbon production and resource recycling (Sadhukhan et al., 2018) has heightened the investigation for PCWPEs (Benedetti et al., 2019; Østby et al., 2022; Li et al., 2024). Concurrently, advanced structure-prediction tools [e.g., AlphaFold (Jumper et al., 2021), Rosetta (Leman et al., 2020), FoldX (Buß et al., 2018), FireProt (Musil et al., 2017, 2021) etc.,] and molecular dynamics simulations (Hospital et al., 2015) have demonstrated robust utility in the precise, mechanism-informed engineering of multiple industrially relevant enzymes (Chowdhury and Maranas, 2020). In light of these advances, this review systematically summarizes key PCWPE engineering strategies developed over the last 10 years, elucidates underlying structure–function principles, and outlines actionable directions for future design and development of industrial PCWPEs.

**FIGURE 1:**
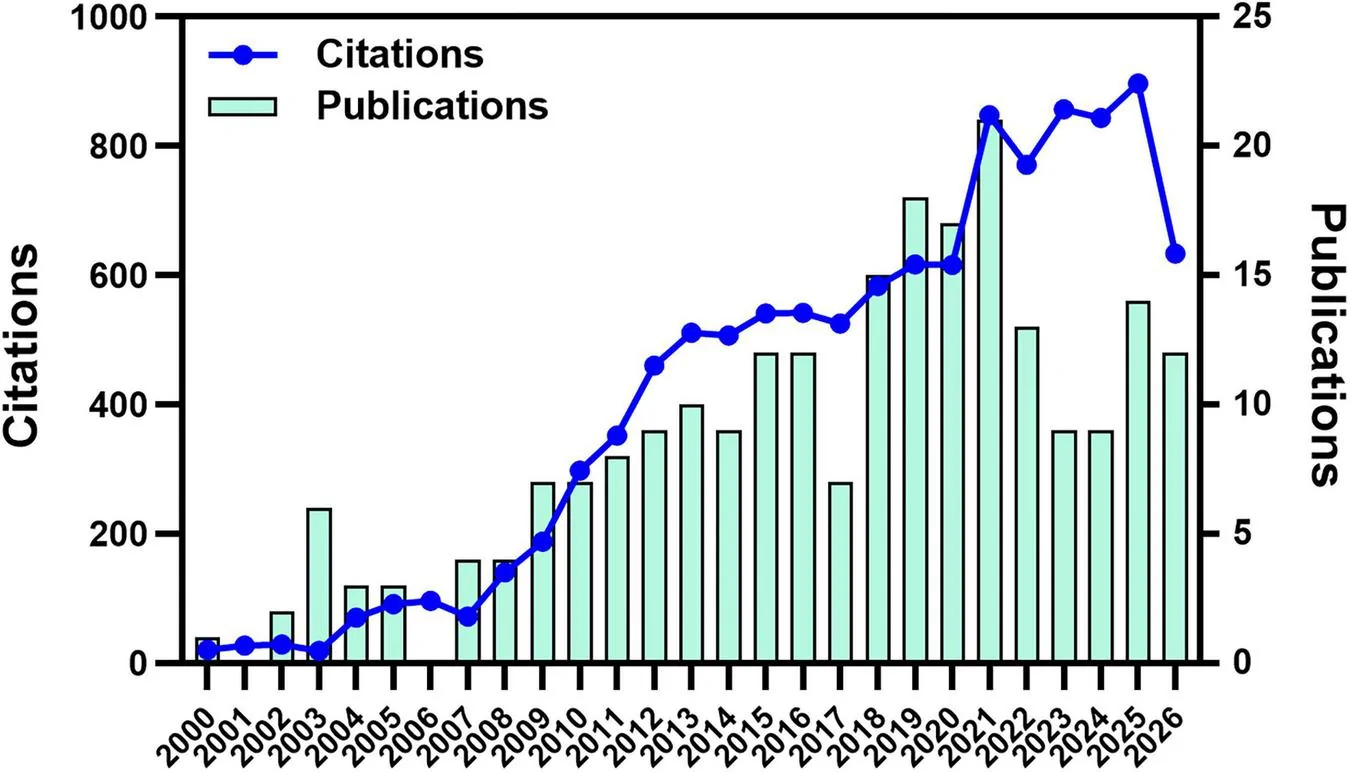
Publication and citation metrics were extracted from Web of Science Core Collection for original research articles and review papers published between year 2000 and 2026 (search date: 21 Sep. 2026). The search query employed was: (“cellulase” OR “hemicellulase” OR “lytic polysaccharide monooxygenase” OR “pectinase”) AND (“directed evolution” OR “rational design” OR “semi-rational design” OR “enzyme fusion”) AND (“microb*” OR “bacteri*” OR “fung*” OR “yeast*” OR “archaea*” OR “actinomycet*” OR “cyanobacter*” OR “protozo*”).

## 2 Industrially mature PCWPEs and their current application status

### 2.1 Cellulase system

The cellulase system comprises endoglucanases (EC 3.2.1.4), exoglucanases (cellobiohydrolases, EC 3.2.1.91), and β-glucosidases (EC 3.2.1.21) (Ejaz et al., 2021). Their complementary activities hydrolyze β-1,4-glycosidic bonds and convert polymeric cellulose to glucose (Xie et al., 2007; Kuhad et al., 2016; de Souza and Kawaguti, 2021), which is why the three enzyme classes are commonly used together in the production of bioethanol from lignocellulosic biomass (Arora et al., 2021). *Trichoderma reesei* represents the most extensively exploited fungi for industrial cellulase production globally, owing to its exceptional native capacity for high-yield secretion of functional cellulolytic enzymes (Bischof et al., 2016). In contrast, bacterial cellulases (e.g., from *Clostridium thermocellum* or *Bacillus* sp.) exhibit enhanced stability under alkaline and thermophilic conditions, rendering them promising candidates for adapting harsh operational environments (Imran et al., 2016). Commercial formulations such as Novozymes’ Cellic^®^ series (e.g., CTec2 and CTec3) can saccharify a range of lignocellulosic feedstocks (Harris et al., 2014). The global cellulase market is projected to grow at a compound annual growth rate (CAGR) of 6.9% and reach USD 3.15 billion by 2032 (Ilić et al., 2023).

### 2.2 Lytic polysaccharide monooxygenase

Lytic polysaccharide monooxygenases are copper-dependent enzymes distinct from classical glycoside hydrolases (GHs) and function as redox-active biocatalysts in biomass conversion (Levasseur et al., 2013). LPMOs are classified as Auxiliary Activities (AA) category of the CAZy database (Frommhagen et al., 2018; Tamburrini et al., 2021). Currently, AA9 family LPMOs, predominantly of fungal origin (e.g., *Trichoderma reesei*) are the dominant class employed industrially for the oxidative degradation of cellulose and hemicelluloses (Johansen, 2016; Frandsen et al., 2021). AA10 enzymes occur in bacteria and eukaryotes, act on chitin or cellulose, and show diverse oxidation patterns and substrate spectra (Chaplin et al., 2016; Pan et al., 2023). Notably, the catalysis of LPMOs depends on a mononuclear copper center, the copper saturation governs enzymatic activity, and incomplete copper loading therefore yields partial inactivation (Østby et al., 2023). The reducing co-substrate and oxidant supply are critical determinants of LPMO turnover: although O_2_ was long assumed to be the sole oxidant, LPMOs exhibit significantly higher turnover rates when using energetically favorable H_2_O_2_ as co-substrate (Hemsworth, 2023). Consequently, optimal LPMO performance necessitates precise regulation of H_2_O_2_ supply to sustain productive turnover while preventing oxidative self-inactivation (Hedison et al., 2021).

The remarkable synergy between LPMOs and cellulases significantly boosts sugar yields by breaking the recalcitrant crystalline regions of microfibrils, which is the primary economic driver for their industrial adoption (Dutta, 2020). This industrial maturity is evident in their established role as a key component of leading commercial enzyme cocktails, such as Novozymes’ Cellic^®^ CTec series, which are deployed in large-scale, commercial facilities for the production of lignocellulosic ethanol (Cannella et al., 2012; Horn et al., 2012). Notably, patents on the use of LPMOs for converting pre-treated plant residues to sugars were filed years before their formal classification, and their subsequent inclusion in enzyme formulations resulted directly from performance improvements observed in industrially relevant assays (Johansen, 2016). Embracing the oxidative cleavage mediated by LPMOs is not merely an academic curiosity but a necessary step toward addressing the planet’s polysaccharide problem and unlocking the full potential of recycling the most abundant polysaccharides (cellulose, hemicellulose, and chitin) (Saraiva Garcia et al., 2026).

### 2.3 β-Xylanase

β-Xylanase (also named as endo-β-1,4-xylanase, EC 3.2.1.8) is a key glycoside hydrolase that catalyzes the endo-type hydrolysis of β-1,4-glycosidic linkages in the xylan backbone, yielding xylooligosaccharides (XOS) of varying degrees of polymerization (Thirametoakkhara et al., 2023). β-Xylanases are widely distributed across bacterial, fungal, and archaeal lineages. Enzymes derived from these phylogenetically distinct sources display marked variation in catalytic properties, thereby constituting a diverse and functionally versatile enzyme resource pool for industrial biocatalysis (Carvalho Andrade et al., 2001; Azzouz et al., 2021; Limsakul et al., 2021). The current industrial landscape for β-xylanases is mature, with well-established commercial utilization across diverse sectors. Their application as feed additives represents the largest market segment, leveraging their ability to hydrolyze anti-nutritional arabinoxylans in animal feed, thereby improving digestibility and growth performance (Fadholly and Aurellia, 2025). The pulp and paper industry is another major consumer, employing β-xylanases for biobleaching, which reduces the need for harsh chlorine-based chemicals (Matos et al., 2024). Furthermore, the food and beverage industry utilizes β-xylanases in baking to improve dough handling and bread quality (Shah et al., 2006), in fruit juice clarification to reduce cloudiness and increase juice yield (Kaushal et al., 2021), and in the production of XOS, which serve as prebiotic ingredients (Santibáñez et al., 2021). The burgeoning biofuel sector also relies heavily on β-xylanases as a key component of enzyme cocktails (e.g., Viscozyme^®^ L) (Santos et al., 2026) for the saccharification of lignocellulosic biomass (Chaudhary et al., 2023). Although there is no precise statistical data, β-xylanases constitute a significant segment of the global carbohydrase market owing to their widespread industrial application (Madhavan et al., 2021).

### 2.4 β-Mannanase

β-Mannanase (EC 3.2.1.78) catalyzes the endo-hydrolysis of β-1,4-glycosidic linkages in the backbones of mannans and galactomannans, yielding prebiotic manno-oligosaccharides (MOS) (Dhawan and Kaur, 2007). This functionality underpins its widespread commercial adoption in the animal feed industry. *Aspergillus* sp. (Kote et al., 2009) and *Bacillus* sp. (Jiang et al., 2006) represent the predominant microbial sources of industrially relevant β-mannanases. As a feed additive, it functions to degrade β-mannans, a type of non-starch polysaccharide (NSP) found in common feedstuffs like soybean meal, copra meal, and guar gum (Kiarie et al., 2021), into MOS, thereby promoting the growth of beneficial gut microflora, while inhibiting pathogenic bacteria (Jang et al., 2024). This further contributes to improved gut health and animal welfare. Hemicell^®^, a ChemGen-developed feed enzyme with high β-mannanase activity, has been widely deployed for this application (European Food Safety Authority [EFSA], 2006). Projections indicate that the global MOS market is projected to grow at a 4.8%–8.5% CAGR from 2025 to 2031 (Karimi et al., 2025), underscoring the continued industrial relevance and growth potential of β-mannanase.

### 2.5 Pectinases

Pectinases refer to a complex group of enzymes that catalyze the degradation of pectic substances. Industrial pectinases are predominantly produced by filamentous fungi, particularly *Aspergillus niger* and *Aspergillus oryzae*, which are favored for their high enzyme yields and efficient secretion (Sarita et al., 2021). Alkaline pectinases, in contrast, are commonly derived from bacterial sources, notably *Bacillus subtilis* strains (Hoondal et al., 2002). Currently, depolymerases represent the most commercially exploited classes that cleave the glycosidic backbone of pectin, reducing its molecular weight and viscosity (Kashyap et al., 2001). They are generally categorized into hydrolases and lyases according to their action mode. Among the hydrolases, endo-polygalacturonases (endo-PGs, EC 3.2.1.15) randomly hydrolyze internal α-1,4-glycosidic bonds in homogalacturonan, whereas exo-polygalacturonases (exo-PGs) sequentially attack the non-reducing ends. Exo-PGs comprise two biochemically distinct subclasses: EC 3.2.1.67 (exo-polygalacturonase) releases galacturonic acid monomers, whereas EC 3.2.1.82 (exo-poly-α-digalacturonosidase) liberates digalacturonic acid units from the terminus, yielding divergent product profiles (Deng et al., 2023). Lyases, by contrast, cleave the α-1,4-glycosidic bond via trans-elimination. Pectin lyases (PNLs/PMGLs, EC 4.2.2.10) target highly methyl-esterified pectin, whereas pectate lyases (PELs/PGLs) prefer de-esterified pectate or low-methoxyl pectin. PELs/PGLs are further divided by cleavage pattern: EC 4.2.2.2 (endolytic pectate lyase) cuts internal bonds, while EC 4.2.2.9 (exolytic pectate disaccharide-lyase) removes unsaturated digalacturonates from the chain end (Zhou et al., 2017). Pectin methylesterases (PMEs; EC 3.1.1.11) remove methyl esters, thereby enhancing pectin’s susceptibility to depolymerases (Li et al., 2021). Notably, rhamnogalacturonases (EC 3.2.1.171) function independently of homogalacturonan degradation but instead degrade the complex, branched rhamnogalacturonan-I domain which represents a critical step in microbial or plant-mediated pectin catabolism (Lemaire et al., 2020).

Food and beverage industry has long been the main application field for microbial pectinases. By the 1960s, pectinases had been maturely adopted for clarification in both fruit juice and wine processing (Kashyap et al., 2001). Additional applications like natural fiber retting, pulp bleaching, animal feed and pretreatment of pectinaceous wastewater, further augment their industrial value (Sarita et al., 2021; Ametefe et al., 2022). Such broad applications have brought worldwide attention to pectinases, which account for approximately 20%–25% of the global enzyme market and project to grow to USD 26.6 billion by 2030 with a CAGR around 11% (Kaul et al., 2024).

## 3 The deficiencies limiting the usage of microbial PCWPEs

The deficiencies observed during the deployment of microbial PCWPEs represent a primary constraint on their functional efficacy, manifesting either as suboptimal catalytic performance under industrially relevant conditions or as severely restricted operational tolerance. The deficiencies fall into five principal categories: (i) insufficient catalytic efficiency constrains the treating efficiency and productivity; (ii) inadequate thermostability renders enzymes incompatible with high temperature processing; (iii) poor adaptability to specific temperature or pH conditions compromises catalytic efficiency and necessitates extensive and costly process optimization; (iv) substrate-related constraints, including lignin interference and structural complexity of plant-derived polysaccharides; (v) product inhibition arising from saccharide accumulation during prolonged biorefinery operations, which attenuates overall enzymatic system performance. Identifying the limitations of microbial PCWPEs in practical applications can provide a scientific basis for their engineering, helping to compensate for these inherent defects and generate variants better suited for industrial use (Adrio and Demain, 2014; Ndochinwa et al., 2024).

### 3.1 Low catalytic efficiency

The low catalytic efficiency of PCWPEs requires high enzyme doses to achieve practical conversion, which results in higher cost. This issue is particularly evident in cellulosic ethanol production, where slow saccharification kinetics limit ethanol yields and fermentation efficiency (Phillips, 2019; Guo et al., 2022). Researchers address this challenge with high enzyme loadings, yet cellulase production cost remains a major economic barrier (Baral et al., 2022), often accounting for 25%–30% of total production costs (Liu et al., 2024). Similarly, in the food and beverage industry, low catalytic efficiency directly limits process throughput, yield, and cost-effectiveness. Suboptimal β-xylanase performance on lignocellulosic feedstocks represents a key bottleneck in XOS production (Carvalho et al., 2013). In juice processing, free pectinases may display limited hydrolysis rates in fruit mashes, directly limiting process efficiency and motivating extensive research into synergistic enhancement strategies, like ultrasound-assisted treatment (Ma et al., 2016; Prajapat et al., 2016). Moreover, in the textile fibers processing, enzymatic degumming offers a greener alternative to energy-intensive alkali treatments (pH 10, 95 °C) (Luo et al., 2024). However, the industrial adoption of enzymatic degumming has been slow, primarily due to the low catalytic efficiency of available enzymes (Wang et al., 2023). This lower efficiency means that enzymatic processes often require longer durations or higher enzyme loadings to achieve a comparable cleanliness to chemical methods (Fan et al., 2021).

### 3.2 Inadequate thermostability

The drive to conduct bioprocesses at high temperatures is rooted in several compelling industrial and economic advantages (Yennamalli et al., 2013; Wohlgemuth et al., 2018). However, native PCWPEs generally exhibit limited thermal stability, undergoing rapid denaturation above their tolerance threshold (Paës et al., 2012; Dadwal et al., 2020; Liu et al., 2023; Chen et al., 2026). Large-scale saccharification often uses elevated temperatures to reduce cellulose-slurry viscosity, improve substrate accessibility, and limit microbial contamination. PCWPEs used under these conditions must remain active and stable (dos Santos Gomes et al., 2019; Liu et al., 2019). The enzymatic activity loss forces operators to either use excessive enzyme loadings to compensate for the loss or accept suboptimal conversion yields. In prebiotic oligosaccharide synthesis, the elevated temperature is also needed to promote the substrate solubility and facilitate the removal of lignin (Ying et al., 2022). However, these thermal requirements severely inactivate high-performance mesophilic enzymes, exemplified by the GH11 β-xylanase XynA from *Streptomyces rameus* L2001 (Wu et al., 2023). Moreover, juice clarification is typically conducted at around 50 °C to achieve optimal enzyme activity and processing speeds (Hosseini et al., 2021). However, even commercially mature pectinases suffer varying degrees of activity loss under these conditions, thereby compromising clarification efficiency (Ortega et al., 2004a,b). Similarly, the feed manufacturing industry commonly uses high temperatures during pelleting, extrusion, and expansion. These conditions pose a challenge for enzyme stability. For example, a study on a commercial enzyme blend containing cellulase, β-glucanase, and xylanase found that over 65% of its activity was lost when subjected to extrusion at high temperatures (Kiarie and Mills, 2019).

### 3.3 Poor adaptability to specific operational scenarios

Certain industrial applications require PCWPEs to function under specific process conditions, typically low-temperatures, non-neutral pH environments and the presence of salts or organic solvents. However, most native PCWPEs are typically adapted to relatively mild catalytic conditions, and exposure to complex operational environments often leads to varying degrees of activity decrease (Liszka et al., 2012). A primary constraint is the discrepancy between catalytic optima and the operational conditions required for specific processes. For instance, β-xylanases often operate optimally at relatively high temperatures (∼60 °C) (Wang et al., 2019; de Oliveira Simas et al., 2024), but these temperatures promote starch gelatinization during dough fermentation which disrupt granule crystallinity and result in poor baking quality. Cooler conditions avoid this issue yet fall below their optima (Lewko et al., 2024). Moreover, pH incompatibility represents another critical limitation. In the pulp bleaching, alkaline conditions (pH > 8.0) represent the predominant industrial operating regime (Lundgren et al., 1994). However, currently studied fungal and bacterial β-xylanases work better under pH 5.0–7.0 (Krengel and Dijkstra, 1996; Sá-Pereira et al., 2002; Gu et al., 2021; Huang et al., 2021), with certain activity loss above pH 8.0 due to ionization changes at active sites (Zhang et al., 2020). In detergent applications aimed at removing fine fibers and preventing fabric pilling, cellulases must remain active across typical washing temperatures (a middle-low range of 20 °C–40 °C) and alkaline conditions (typically pH 8.0–10.5 in laundry detergents) (Behera et al., 2017). However, commercial fungal cellulases are commonly optimized for approximately 50 °C and acidic to mildly acidic pH (typically pH 4.5–6.0), making them poorly suited for direct use in laundry detergents (Boer and Koivula, 2003; Onofre et al., 2015; Herlet et al., 2017; Malik and Javed, 2021). The intrinsically acidic nature of many fruit juices also compromises the performance of some commercial pectinases during the clarification, as their optimal catalytic pH typically falls within the neutral to mildly acidic range (pH 5–7) (Prajapati et al., 2021; Kaissar et al., 2025). Some process settings place unusual demands on enzyme performance. Large biorefineries consume substantial amounts of water, and seawater can be a cost-effective alternative to potable water; its high salinity, however, may reduce PCWPE activity (Domínguez and de María, 2013). Similarly, organic solvents used in lignocellulosic biomass pretreatment to remove lignin and hemicelluloses can enhance cellulose accessibility (Vaidya et al., 2022), but residual solvents may disrupt the active conformation of an enzyme and reduce its activity (Kim et al., 2011).

### 3.4 Hard to process complex plant-based materials

The conformations and tailored active cavities of PCWPEs allow them to recognize particular glycans. Industrial biomass, however, contains substrates with complex compositions and structures (da Silva et al., 2025). Lignocellulosic biomass is the most abundant renewable resource in nature and a key material for biorefinery (Yuan et al., 2021; Hu et al., 2023). Its composition varies across agro-wastes (wheat straw: 35.0%–39.0% cellulose, 23.0%–30.0% hemicellulose, and 12.0%–16.0% lignin; sugarcane bagasse: 25.0%–45.0% cellulose, 28.0%–32.0% hemicellulose, and 15.0%–25.0% lignin) (Isikgor and Becer, 2015). The high lignin content and compact structural organization of lignocellulosic biomass impede enzymatic saccharification (Ravindran and Jaiswal, 2016). Fungal-secretome cocktails are common in biorefineries (Pimentel et al., 2021; Raulo et al., 2021), but they are not readily tailored to individual substrates, and their multienzyme interactions still require optimization (Van Dyk and Pletschke, 2012; Bianco and Perrotta, 2015). Lignin can irreversibly adsorb cellulase components through nonspecific interactions, hydrogen bonding, and electrostatic forces. Such non-productive binding diverts these enzymes from their target polysaccharide sites, reducing the available concentration of active enzymes (Gao et al., 2014). Branched, heterogeneous polysaccharides add another constraint because their composition and structure can reduce the effectiveness of PCWPE treatment. For instance, arabinoxylan features a β-(1→4)-linked xylan backbone variably substituted with α-L-arabinofuranose residues at O-2 and/or O-3 positions, which can be further esterified with ferulic acid (Huang et al., 2024). Similarly, the pectin hairy regions exhibit substantial complexity. Rhamnogalacturonan-I (RG-I) features a rhamnose-galacturonic acid backbone substituted with variable arabinan and galactan side chains (Kaczmarska et al., 2022), whereas Rhamnogalacturonan-II (RG-II), though smaller, decorates with a highly complex array of side chains containing rare sugars (Barnes et al., 2021). The complexity of above-mentioned substrates has brought about additional difficulties for the effective application of PCWPEs.

### 3.5 Product inhibition

Certain PCWPEs frequently encounter product inhibition caused by the accumulation of small oligosaccharides or monosaccharides during long-term biorefinery, particularly occurring in cellulase system (Bhatia et al., 2024). Cellobiose, the hydrolytic product of cellobiohydrolases (CBHs), competitively inhibits both endoglucanases (EGs) and CBHs by blocking their active sites. GH7 family CBHs are most sensitive ones to such type inhibition, followed by GH6 family CBHs and EGs (Brown et al., 2024). Representatively, the *IC*_50_ for *Trichoderma reesei*-derived CBHs *Tr*Cel7A and *Tr*Cel6A toward cellobiose are 0.38 mM and 16 mM under 25 °C when acting with homologous EG (*Tr*Cel5A), thus indicating prominent susceptibilities (Teugjas and Väljamäe, 2013). Moreover, β-glucosidases (BGLs), which convert cellobiose to glucose, undergo feedback inhibition at elevated glucose levels. This inhibition can disrupt the interaction between CBHs and BGLs, thereby diminishing the overall hydrolysis efficiency (Costa et al., 2019). Similarly, the primary products of xylan hydrolysis like xylose and arabinose, inhibit β-xylanases and β-xylosidases (Rohman et al., 2018). For instance, D-xylose competitively inhibits β-xylosidases, with significant activity loss observed at concentrations ≥ 100 mM (Rohman et al., 2019). The β-xylosidase from *Talaromyces emersonii* was reported to be especially sensitive with an apparent *K*_*i*_ of 1.3 mM (Rasmussen et al., 2006). Pectin-degrading enzymes face similar problems: unsaturated oligogalacturonides (e.g., Δ4,5-galacturonides), products of pectate lyase, can also inhibit polygalacturonases by competing for active sites (Satapathy et al., 2020).

## 4 Specific engineering strategies for industrially relevant PCWPEs to overcome inherent performance limitations

Engineering industrially relevant PCWPEs is difficult, primarily because these enzymes act on complex polysaccharide substrates (Cragg et al., 2015). These intricate substrates have driven PCWPEs to evolve into more elaborate structures. Unlike traditional industrial enzymes like proteases, lipases, and α-amylases whose substrates are relatively simple, most PCWPEs exhibit multi-modular organizations or specialized structural features. Cellulases and xylanases frequently contain carbohydrate-binding modules (CBMs) in conjunction with catalytic domains (CDs), facilitating the binding of crystalline cellulose or hemicellulose within lignin-rich plant cell walls (Boraston et al., 2004). Some β-xylanases even incorporate a deacetylase domain (NodB) to remove acetyl groups from xylan, enhancing backbone cleavage (Laurie et al., 1997). Certain pectinases feature large, complicated parallel beta-helix motifs with irregular loops, enabling them to process pectin substrates with varying degrees of methylation (Kanungo and Bag, 2019). Such structural complexity poses significant challenges in identifying suitable modification sites and developing effective engineering strategies. Furthermore, the bioconversion of complex plant-based materials, like lignocellulosic biomass, typically requires the synergistic action of multiple PCWPEs (Puentes-Téllez and Falcao Salles, 2018). Within such enzyme systems, individual components are highly likely to exhibit distinct functional deficiencies, necessitating tailored engineering approaches to enhance inter-enzyme synergy. Enzymes exhibiting low catalytic efficiency can become rate-limiting in the overall conversion process, while those susceptible to product inhibition may cause accumulation of intermediates or end products, thereby compromising efficiency of entire system (Van Dyk and Pletschke, 2012). Additionally, enzymes prone to non-productive binding with lignin components in agro-waste are effectively depleted from the reaction system, reducing their available concentration and compromising catalytic performance (Tokunaga et al., 2020). Given these complexities, the selection of appropriate engineering targets and strategies is crucial for advancing PCWPE applications. Therefore, a systematic summary, critical analysis, and identification of promising research directions based on current advancements are essential. Representative studies on PCWPE engineering over the past decade are summarized in Supplementary Tables 1–6.

### 4.1 Improving catalytic efficiency

Greater catalytic efficiency offers potential for reducing enzyme dosage and operational costs if the gain persists under process-relevant conditions. However, current engineering efforts for higher catalytic efficiency remain largely confined to proof-of-concept studies conducted under controlled laboratory settings, using well-defined model substrates (e.g., microcrystalline cellulose). This limitation is due to persistent challenges, including substrate heterogeneity, difficulty in precisely controlling industrially applicable conditions, and the absence of high-throughput, scale-relevant activity assays. Catalytic performance depends on substrate access, binding, catalytic conversion, and product release within the active-site cleft (Hammes et al., 2011). Therefore, this region represents a primary target for engineering efforts aimed at enhancing efficiency. However, the development of a universal strategy for enhancing PCWPEs’ efficiency remains elusive, primarily due to the diverse substrate recognition modes and catalytic pathways exhibited by different members of this enzyme class.

Currently, introducing tailored modifications around substrate-binding sites represents a widely adopted strategy for enhancing catalytic efficiency (see Supplementary Table 1 for representative examples). Lv et al. (2024) improved the catalytic efficiency of a GH5 processive endoglucanase EG5C-1 from *Bacillus subtilis* BS-5 by combining consensus analysis with the optimization of substrate-binding loop (Gly230-Phe241) guided by MD simulations. The final combinatorial mutant K91I/A198T/Q237D/V240P exhibited 8.5- and 4.8-fold increases in catalytic efficiency (k_*cat*_/K_*m*_) against the sodium carboxymethylcellulose (CMC-Na) and phosphoric acid-swollen cellulose (PASC) (Lv et al., 2024). LPMOs are of interest for processing recalcitrant lignocellulosic biomass. However, relevant engineering efforts remain limited. LPMO activity assays require precise condition control (peroxide supply, copper loading etc.), so we provide full experimental details for clarity. Berhe et al. (2023) engineered BaLPMO10A from *Bacillus amyloliquefaciens* using consensus analysis alone. Activity was detected toward chromogenic 2, 6-dimethoxyphenol (2,6-DMP), using Cu^2+^-saturated enzyme (0.5 μM, pre-incubated with a fourfold molar excess of Cu^2+^) with 100 μM H_2_O_2_ as the co-substrate. The best performed V40L/E124D showed up to a 93.7% increase in activity compared with wild-type (Berhe et al., 2023), however, the study offered little structural interpretation. The structure-function basis for engineering LPMOs remains less developed than that for established glycoside hydrolases. Lai et al. (2020) designed 47 mutants of Xyn30Y5, a β-xylanase sourced from *Bacillus* sp. 30Y5, by combining eight rational strategies. The final mutant 3B (G229S/M225I/V245L/D293Q/D322N) showed a 3-fold increase in specific activity and doubled catalytic efficiency. MD simulation revealed that the flexibility enhancement of α5- helix and loop7 located near to the catalytic residues might facilitate the catalysis and substrate binding, thus leading to higher catalytic efficiency (Lai et al., 2020). Sun et al. (2022) rationally reshaped the substrate-binding cavity of a β-mannanase PcMan113 derived from *Paenibacillus cineris*. The F110E/N246Y double mutant showed a 5.5-fold increase in k_*cat*_/K_*m*_ toward mannopentaose. The authors proposed that both substitutions reduce steric hindrance to substrate access (Sun et al., 2022). Wang et al. (2018) engineered an alkaline pectate lyase PEL168 from *Bacillus subtilis* 168 through multiple methods. Firstly, the structure-guided method found a single mutant V132F which exhibited a 1.7-fold activity increase. Secondly, K47E was identified by directed evolution, showing better substrate affinity and higher turnover number. Thirdly, the residue at site 47 was optimized by saturation mutagenesis on K47E/V132F and the mutant K47D/V132F displayed a 3.9-fold activity increase to polygalacturonic acid (PGA). Finally, R272W near catalytic residue Lys278 was superimposed on K47D/V132F, constructing the triple mutant K47D/V132F/R272W, achieving a 4.0-fold activity increase (5610 U/mg with 1 mM Ca^2+^). The structural interpretation indicated that the interaction changes between specific residues in the triple mutant produced a larger, more open catalytic cleft that facilitated substrate binding and product release (Wang et al., 2018).

Considered alongside the representative studies in Supplementary Table 1, these findings suggest three established strategies for enhancing the catalytic efficiency of PCWPEs (Figure 2): (i) Enlarging the active site cavity or reducing steric hindrance to improve substrate access and product release; (ii) Adjusting functional residues or local flexibility around the binding site; and (iii) Fusing CBMs or substitution of key structural regions (e.g., the N-terminus of β-xylanases) with corresponding segments from highly efficient homologues to obtain enhanced catalytic efficiency. However, strategy (i) lacks a unifying mechanism and exhibit limited coherence. In contrast, strategy (ii) lacks rigorous structural interpretation (Li et al., 2017; Xue et al., 2025). As PCWPEs belong to the broader class of carbohydrate-active enzymes (CAZymes), rational design based on the shared structural features and functional group distribution of polysaccharide substrates could establish a universal strategy for engineering industrially relevant PCWPEs.

**FIGURE 2:**
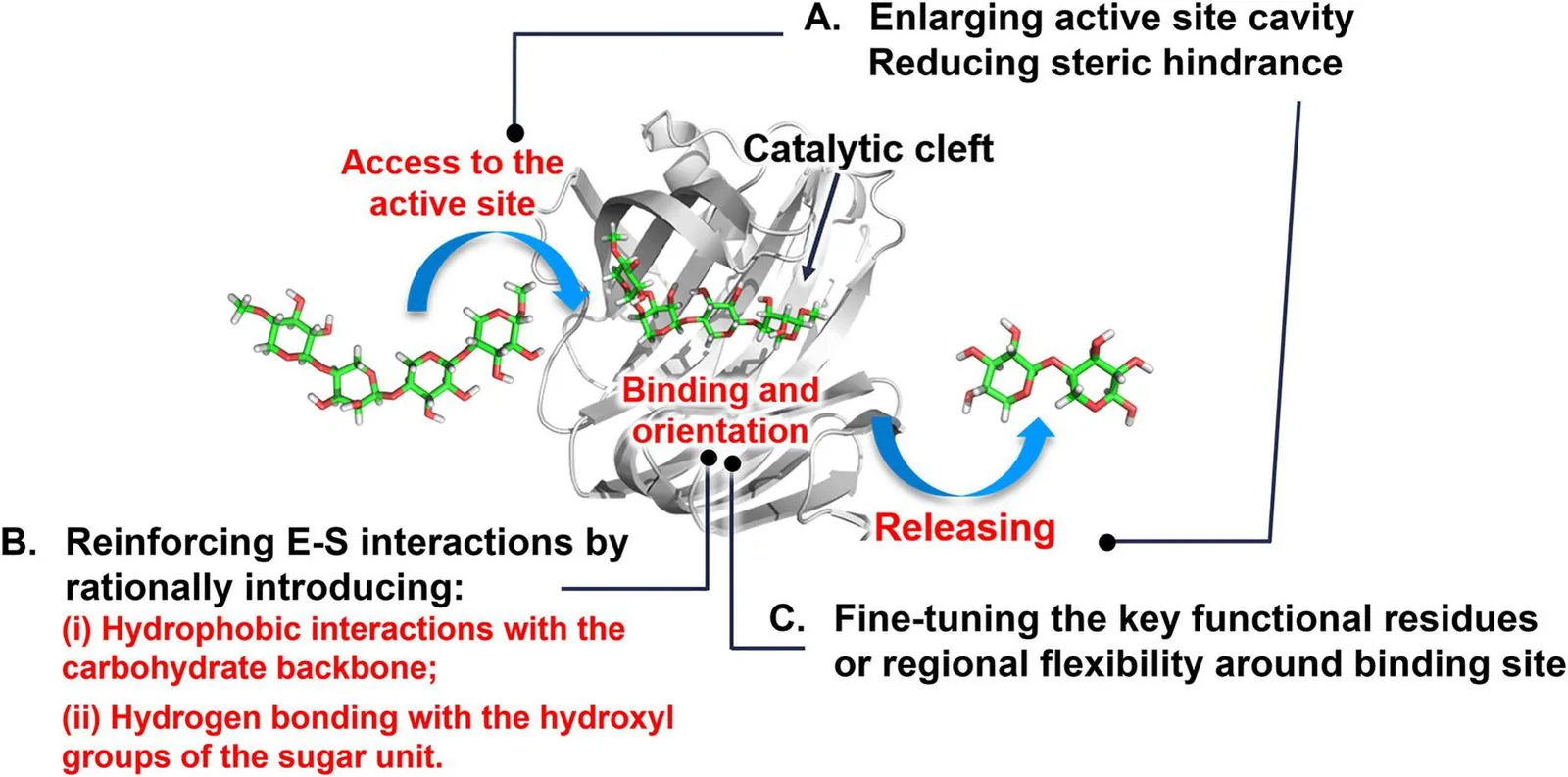
Proposed strategies for improving catalytic efficiency focus on three aspects: **(A)** Enlarging the catalytic cavity or reducing steric hindrance to facilitate substrate access and product release; **(B)** Reinforcing enzyme-substrate interactions to optimize binding; **(C)** Fine-tuning the structure characteristics around binding site. The enzyme model used here is the catalytic domain of GH11 β-xylanase Xyn1G3 (AlphaFold DB accession ID: AF-G9BYA7-F1). Fusing CBMs or replacing other key regions were not included in this figure for the insufficient structure interpretation in the relevant works. See Supplementary Table 1 for relevant studies.

In addition to above strategies, we have also noticed that reinforcing hydrophobic interactions and hydrogen bonds are key determinants for catalytic efficiency enhancement of PCWPEs (Figure 2). Firstly, the hydrophobic packings of aromatic residues with carbohydrate backbone are pivotal for anchoring bulky polysaccharide substrates, as indicated by the frequent occurrence of aromatic residues in catalytic cavity (Acquah et al., 2018). Our group discovered that Phe226 (+2 subsite) and Phe414 (−1 subsite) in the active cavity of a *Thermotoga naphthophila*-derived β-glucosidase *Tn*0602 are crucial for substrate recognition by hydrophobic packing. Mutations like F226G and F414S only lead to dramatic loss of hydrolytic activity (Yang et al., 2017; Lyu et al., 2022). Furthermore, studies on processive cellulases (e.g., EGs and CBHs) reveal that their active tunnels are lined with aromatic residues acting as “molecular lubricants,” facilitating the sliding of the cellulose chain through the tunnel and reducing the energetic barrier to processivity (Chaudhari et al., 2023). Secondly, hydrogen bonds formed between polar residues (Asp/Glu, Lys/Arg, Asn/Gln) and hydroxyl groups on polysaccharide can improve substrate recognition and transition-state binding, ensuring precise substrate orientation and efficient cleavage (Pramanik et al., 2021). For instance, hydrogen bonding networks involving residues such as Asp/Glu and Asn/Gln facilitate tight binding and catalytic precision, as demonstrated in various CAZymes (Xie et al., 2001; Beckham et al., 2010; Kikuchi et al., 2017; Xi et al., 2023; Chen et al., 2025). To sum up, rational engineering targeting hydrophobic interactions and hydrogen bonds leverages fundamental molecular mechanisms of PCWPEs, which can be an effective strategy for enhancing catalytic efficiency.

### 4.2 Enhancing thermostability

As depicted in Figure 3 and Supplementary Table 2, recent strategies for enhancing thermostability of PCWPEs are highly similar. Most researchers employed rational design, which typically consists of two steps. Firstly, the flexible regions are identified by MD simulation or B-factor analysis. Secondly, the residues with stabilizing effect are introduced based on *in-silico* aided methods or consensus sequence analysis with homologous enzymes.

**FIGURE 3:**
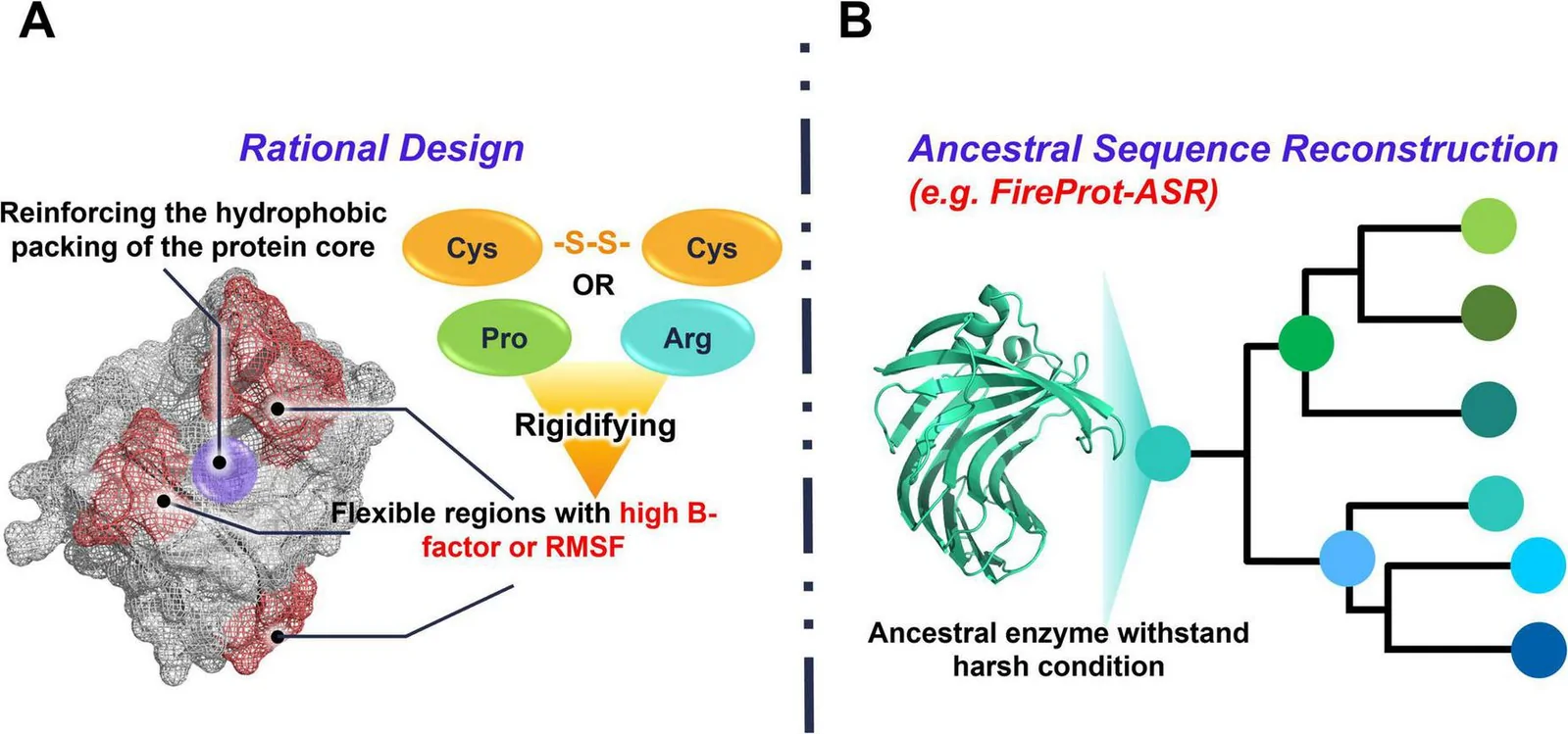
Proposed strategies for enhancing thermostability. Prevailing approaches include: **(A)** stabilizing the flexible regions identified by MD simulation or B-factor analysis by introducing stabilizing mutations or reinforcing the hydrophobic packing of the protein core; **(B)** ASR provides a new avenue to identify ancestral enzymes that can withstand harsh condition. The enzyme model used here is GH11 β-xylanase XynA (AlphaFold DB accession ID: AF-A0A1S2NZZ1-F1). See Supplementary Table 2 for relevant studies.

Notably, Gao et al. (2023) conducted a comprehensive loop optimization of a *Trichoderma reesei* endoglucanases (EG) through machine learning, representing a rare example. Guided by B-factor analysis, specific residues (Q180 and N287 of EGI, Gly402 and Ser404 of EGII) detrimental to thermostability were singly mutated based on machine learning models trained on 350,000 existing primary sequence datasets. Consequently, single mutants EGI-N287I and EGII-S404H showed approximately 3-fold higher retaining activities under 70 °C treatment for 30 min (Gao et al., 2023). Zhou et al. (2022) stabilized a chitin-active LPMO, *Cj*LPMO10A from *Cellvibrio japonicus*, by rationally introducing disulfide bonds. Combining B-factor analysis with assessment of disulfide bond forming sites, two residue pairs (Asn78-His116 and Asp59-Arg121) distal to the substrate-binding surface were mutated into cysteine to introduce disulfide bonds. Residue activity in thermal inactivation assay was measured using 2.6-DMP as substrate and Cu^2+^-saturated enzyme (0.1 mg/mL, pre-incubated with a fourfold molar excess of Cu^2+^) with 100 μM H_2_O_2_ as the co-substrate. The best variant M1 (N78C/H116C) exhibited a 3-fold longer half-life at 60 °C, a 3.5 °C increase in *T_50_^15^*, and a 7.0 °C rise in apparent *T*_*m*_. RMSF analysis revealed that N78C/H116C greatly reduced flexibility of targeted loops, thereby enhancing the overall thermostability (Zhou et al., 2022). Wu et al. (2023) identified several N-terminal loops of XynA, a β-xylanase derived from *Streptomyces rameus* L2001, as key regions influencing thermostability through MD simulations. Potential residues were optimized based on multiple sequence alignments with homologues exhibiting excellent thermostabilities. The final mutants, Mut1 and Mut2, were constructed by rationally combining the mutations loop2-^11^YHDGYF^16^, loop3-^23^AP^24^/^23^SP^24^, and loop4-^32^GP^33^ could maintain residual activity exceeding 85% after incubation at 80 °C or 90 °C for 12 h (Wu et al., 2023). Tan et al. (2023) improved the thermostability of a β-mannanase *An*man from *Aspergillus niger* CBS513.88 by modifying loop regions with higher B-factor on the protein surface. Subsequent virtual saturation mutagenesis was employed to screen for potential mutants with decreased ΔΔG values. After combination, the final mutant, Mut5, carrying the proline substitutions (E15C/S65P/A84P/A195P/T298P), showed a 7.8-fold prolonged half-life compared to the wild-type at 70 °C (Tan et al., 2023). Zhou et al. (2022) engineered the thermo-alkaline stability of an alkaline pectate lyase from *Bacillus clausii* S10 by semi-rational design. Target sites were selected based on high B-factors in loop and turn regions, and potential stabilizing mutations were predicted *in-silico* using HoTMuSic^1^ (Pucci et al., 2020) to confirm their impact on the melting temperature (Δ*T*_*m*_). Subsequent mutant library was screened by activity detection under 70 °C, pH 10.5. The triple mutant R150G/R216H/A238C showed an 8.5 °C increase in the *T_50_^15^* value at pH 10.0 and 1.8-fold higher specific activity at 60 °C (Zhou et al., 2022).

To sum up, directed evolution coupled with screening under elevate temperature constitutes a robust, canonical method (Goedegebuur et al., 2017; Cao et al., 2018), whereas reinforcing the flexible loops or turns offers a more rational and efficient alternative (Figure 3A). It is noteworthy that loop engineering has the potential to alleviate the entropic penalty associated with protein folding. Moreover, it avoids perturbing the active conformation of the catalytic pocket, thereby offering a more effective strategy to counteract the stability-activity trade-offs often encounter in stability engineering (Gao et al., 2023; Wu et al., 2023). Furthermore, reinforcing the hydrophobic packing of the protein core and Ancestral Sequence Reconstruction (ASR) are also effective strategies for enhancing thermostability. As summarized in Figure 3A, enhancing the hydrophobicity of buried residues to improve hydrophobic packing within the protein core can strengthen overall structural rigidity, thereby increasing the enzyme’s tolerance to elevated temperatures (Eijsink et al., 1992; Lee et al., 2017). Moreover, new tools like ASR can leverage insights into evolutionary relationships and enable the revival of ancient enzymes (Figure 3B), which often exhibit superior thermostability, broader pH tolerance, and enhanced catalytic efficiency compared to their modern homologues (Livada et al., 2023; Wang et al., 2024). A representative web server, FireProt-ASR^2^ can automatically calculate ancestral sequences, facilitating the identification of candidates that can withstand harsh conditions (Musil et al., 2021).

### 4.3 Optimizing adaptability to specific industrial scenarios

To date, various industries impose specific requirements on PCWPEs. Conditions like low-temperatures, non-neutral pH, and the presence of salts or organic solvents often reduce enzymatic performance. However, mechanistic studies on engineering solutions remain limited. Fine-tuning PCWPEs for specific operational conditions through precise engineering will fulfill the potential of current well-studied enzymes, enabling reduced enzyme loading, improved treatment efficiency, and extended enzyme functional longevity.

#### 4.3.1 Low-temperature adaptability

The low-temperature adaptability of PCWPEs has received limited attention from researchers, only You et al. (2021) have attempted to identify the key sites for enhancing the low-temperature catalytic efficiency in β-xylanase XYL10C_ΔN, a truncated β-xylanase derived from *Bispora* sp. MEY-1. They discovered that mutating Asn207 to the more flexible glycine residue resulted in a mutant (N207G) with increased structural flexibility and higher activity at medium and low-temperatures (≤40 °C) (You et al., 2021).

Psychrophilic enzymes provide a mechanistic blueprint for low-temperature adaptability, which can be achieved through structural features that are antagonistic to those conferring thermostability, such as increased conformational flexibility and a less densely packed hydrophobic core. Sonan et al. (2007) demonstrate that the unusually long, hydrophilic, and flexible linker region (LR) connecting the catalytic module (CM) and carbohydrate-binding module (CBM) in the psychrophilic cellulase Cel5G is crucial for its low-temperature adaptability. Shortening this LR increased thermostability but reduced low-temperature activity (Sonan et al., 2007). Tang et al. (2019) identified the cold-active pectate lyase PEL1 from an Antarctic bacterium *Massilia eurypsychrophila* and found that the Pro and Arg contents of this enzyme were relatively low among its homologues. Meanwhile, it featured more random coils and fewer β-sheets. These characteristics all reveal the underlying basis of cold adaptation (Tang et al., 2019). Zhang et al. (2021) reported that the psychrophilic acetyl-xylan esterase AlAXEase from *Arcticibacterium luteifluviistationis* SM1504^ T^ possessed the longest catalytic loop compared to homologues. The absence of stabilizing interactions within regions like ^192^-KDRG-^195^ grants this loop greater flexibility, facilitating substrate binding/catalysis in the cold (Zhang et al., 2021). Extensively studied cold-adapted lipases also provide valuable references into structural determinants of low-temperature adaptability, including reduced Arg and Pro contents, diminished hydrophobic core packing, fewer salt bridges, and attenuated aromatic-aromatic interactions (Alquati et al., 2002; Wang et al., 2021; Yang et al., 2021). Therefore, a targeted destabilization strategy that increases structural flexibility rather than enhancing stability or rigidity (Section “4.2 Enhancing thermostability”) represents an effective approach to improve the catalytic activity of PCWPEs under low-temperature conditions, consistent with the known stability-flexibility trade-off in cold-adapted enzymes (Collins and Feller, 2023). Potential targets for enhancing conformational flexibility include the active-site cavity, substrate-recognition domains, or interdomain linkers. Further engineering approaches are illustrated in Figure 4A: (i) Weakening the hydrophobic packing of the protein core to decrease the compactness of the protein interior (Gianese et al., 2002); (ii) Introducing small, flexible residues (e.g., Gly, Ser) to provide extra local mobility (Huang et al., 2023); (iii) Moderately extending loops or interdomain linkers to increase conformational flexibility (Sonan et al., 2007; Miao et al., 2016); (iv) Rationally substituting rigid Pro residues in loops with more flexible residues (Balaei et al., 2025); (v) Strategically substituting Arg residues with Ala to reduce ionic or hydrogen bonding and enhance local flexibility (Dachuri et al., 2022). MD simulations provide a robust tool for verifying the impact of the above modifications, thus preventing negative effects on the functional conformation (Huang et al., 2024).

**FIGURE 4:**
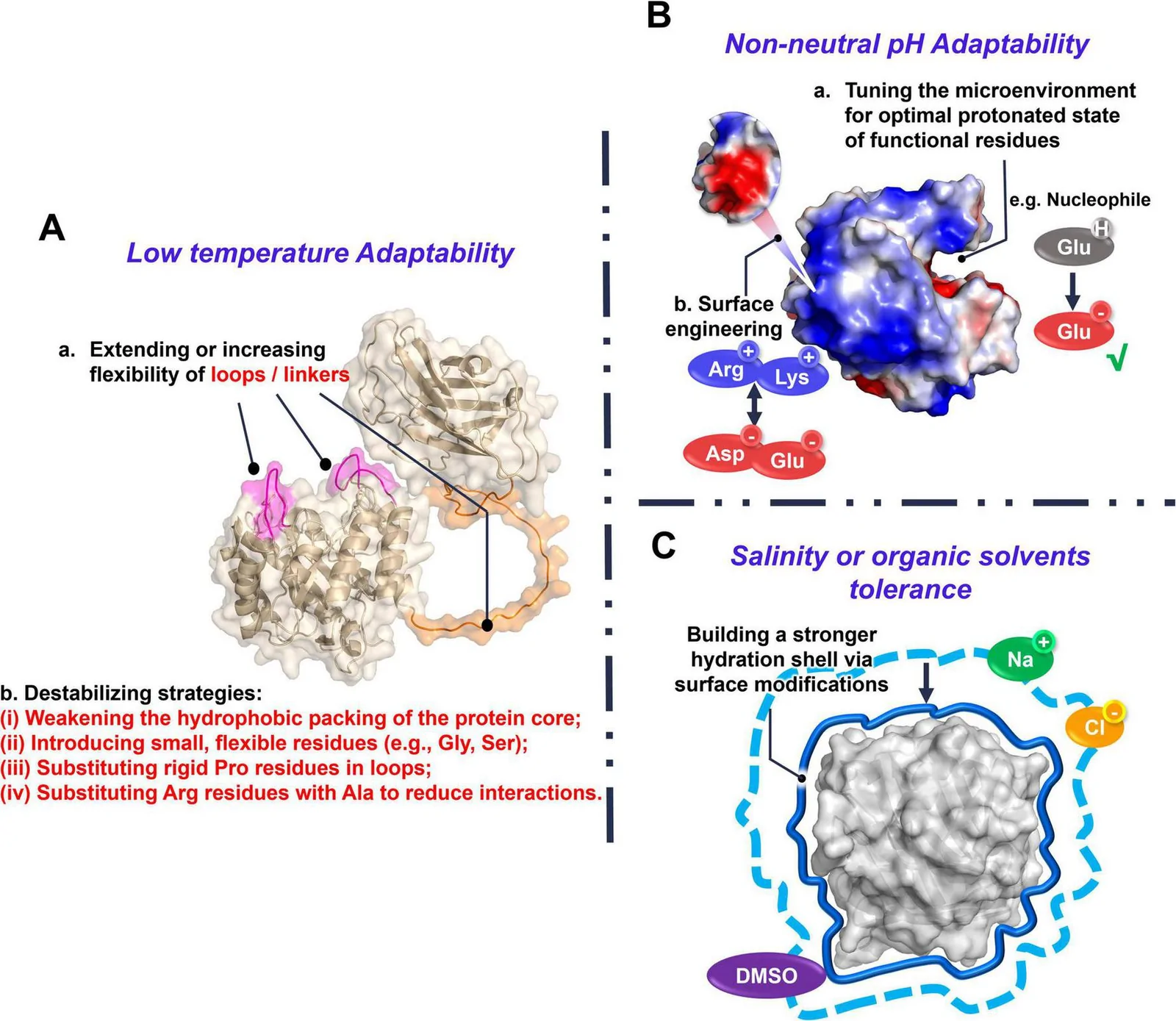
Proposed strategies for enhancing adaptability to specific industrial scenarios: **(A)** low-temperature adaptability can be achieved by extending loops/linkers or introducing destabilizing modifications (enzyme model: GH5 endoglucanase EglS, AlphaFold DB accession ID: AF-P10475-F1); **(B)** non-neutral pH adaptability can be optimized by surface engineering or fine-tuning the microenvironment of active site (enzyme model: GH11 β-xylanase XynB, AlphaFold DB accession ID: AF-P17137-F1); **(C)** tolerance to high salinity or organic solvents can be enhanced by reinforcing hydration shell (enzyme model: GH11 β-xylanase XynB, AlphaFold DB accession ID: AF-P17137-F1). See Supplementary Table 3 for relevant studies on pH adaptability.

#### 4.3.2 Non-neutral pH adaptability

The pH adaptability of enzymes is determined by three main factors. First, evolutionary origins or hosts dictate their inherent pH optima (Koivulehto et al., 2020). Second, the protonation or deprotonation states of ionizable residues (e.g., Asp, Glu, His, Lys) at active sites affect charge distribution and affecting substrate affinity (Wu et al., 2020). Third, salt bridges and hydrogen bonds formed by these ionizable residues play critical roles in maintaining structural conformation within the functional pH range (Evan et al., 2020).

Representative works summarized in Supplementary Table 3 indicate that the surface engineering, particularly charge tuning, appears to be a promising strategy. Xia et al. (2016) broadened the operational pH range of a β-glucosidase Bgl3A from *Talaromyces leycettanus* by leveraging comparative sequence-structure analysis with the pH-tolerant homologue *Nf*BGL1 from *Neosartorya fischeri* P1 (73% identity). Removal of O-glycosylation sites yielded mutants M1 and M2, which exhibited enhanced pH tolerance across a broad range (pH 3.0–10.0). This improvement was likely attributable to change in spatial structure, though the underlying electrostatic alterations remained uncharacterized (Xia et al., 2016). Xiang et al. (2019) performed directed evolution of β-xylanase XynHBN188A from *Bacillus pumilus* HBP8. The N42D mutation confers enhanced enzymatic performance under acidic conditions, likely attributable to the introduction of a negatively charged carboxyl group at position 42 and thereby modulating the local electrostatic surface potential. Similarly, in the directed evolution of a β-mannanase *Rm*Man5A derived from *Rhizomucor miehei* CAU432, critical mutations Y233H and K264M transformed the weak protein surface negative charge into stronger state, thereby improving the acidic adaptability (Li et al., 2017). Using Rosetta Supercharge predictions, Li et al. (2024) engineered the surface of a chimeric pectin lyase CpelA created by fusing CBM9-2 from *Thermotoga maritima* to the N-terminus of *Aspergillus niger* PelA. The M6 variant (S514D/S538E) shifted the optimum pH from 6.0 to 4.0, increased activity 2.8-fold, and improved stability at pH 3.0. Such surface modification may enhance structural rigidity and substrate accessibility under acidic conditions (Li et al., 2024).

Based on the underlying patterns, pH adaptability can be tuned through protein surface engineering or modifying the active site (Figure 4B). Surface engineering principally operates through the rational introduction, removal, or substitution of charged residues (e.g., Asp, Glu, Lys and Arg) to modulate local charge distribution, thus maintaining structural integrity across pH extremes (Liu et al., 2008; Qiu and Lai, 2013; Feng et al., 2025). As for the active site, fine-tuning the microenvironment can reconfigure the electrostatic network (Nielsen et al., 1999) and hydrogen-bonding patterns (Joshi et al., 2000). These modifications optimize the protonation states of functional residues [e.g., in retaining GH enzymes, the γ-carboxyl group of nucleophilic glutamate residue acts in its deprotonated state (Soliman et al., 2009)], thereby facilitating the catalytic process and enhancing tolerance to pH fluctuations (Li et al., 2022). The complexity of pH adaptability arises from the interplay of these factors, making the development of a single, predictive strategy highly challenging. Advanced machine learning approaches are required to gain deeper mechanistic insights both considering surface charge and protonation of key residues.

#### 4.3.3 Enhancing tolerance toward salinity and organic solvents

High salt environments and organic solvents can disrupt the hydration shell, hydrogen bonding network, and electrostatic interactions of enzymes, thereby altering their active conformation and leading to activity loss. Currently, the primary strategy to address this challenge in PCWPEs is screening promising candidates from extremophiles (Li et al., 2012; Amoozegar et al., 2019). These enzymes inherently exhibit high stability and are thus more likely to tolerate extreme conditions. Although engineering of salt and solvent tolerance in PCWPEs has seen limited progress, studies on other enzymes offer promising avenues. In order to increase saline tolerance, Warden et al. (2015) engineered a well-studied bovine carbonic anhydrase II by replacing surface residues (mostly non-ionizing) with acidic Asp and Glu in three stages. The final mutant, M4, with 18 mutations, even retained strong activity in 3 M NaCl. This design strengthens interactions with the ordered first Na^+^-hydration shell around the protein surface, conferring high salt tolerance (Warden et al., 2015). Cui et al. (2021) disrupted three unfavorable salt bridges on the surface of *Bacillus subtilis* lipase A (BSLA), identified by MD simulations in water and three organic solvents (1,4-dioxane, dimethyl sulfoxide, 2,2,2-trifluoroethanol). The variant D64K/D144K, obtained through iterative mutagenesis, showed up to 7.6-fold higher resistance to the solvents. Notably, the variants exhibit slightly stronger hydration shells overall, especially around the uncaged charged residues, which is key to their enhanced organic solvent tolerance (Cui et al., 2021). Hence, maintaining a strong hydration shell around the protein surface (Figure 4C) by enhancing interactions between surface residues and water molecules helps preserve the enzyme’s solvated state, resulting in higher tolerance to salts and organic solvents.

### 4.4 Improving processing capacity toward plant-based materials

Efficient processing of complex plant biomass typically necessitates synergistic combinations with complementary functions (Van Dyk and Pletschke, 2012). Meanwhile, minimizing non-productive binding to lignin is essential to maintain a higher effective enzyme proportion (Huang et al., 2022). As summarized in Supplementary Tables 4, 5, this processing capacity can be improved by protein engineering through two main ways: (i) Employing enzyme fusion to place complementary activities in one construct; (ii) Weakening the lignin affinity in specific regions. Relevant approaches are also illustrated in Figure 5.

**FIGURE 5:**
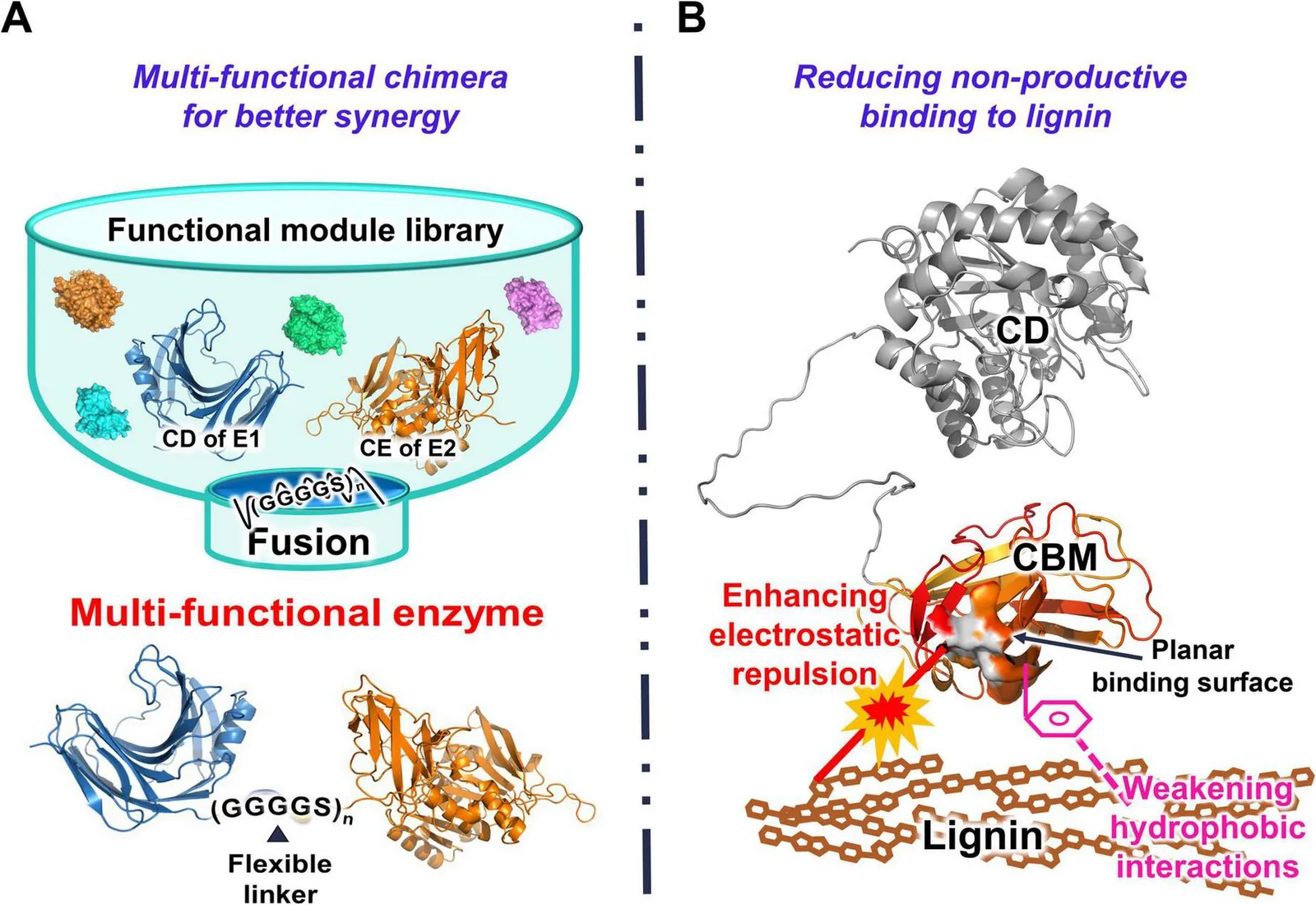
Proposed strategies for improving processing capacity toward complex plant-based materials: **(A)** creating multi-functional chimera for improved synergy based on tailored functional module library (CD of E1: GH11 β-xylanase XynII, PDB ID: 1YNA; CE of E2: Feruloyl esterase domain of GH10 β-xylanase Xyn-Fae, AlphaFold DB accession ID: AF-D5EY13-F1); **(B)** reducing non-productive binding to lignin can be achieved by perturbing intermolecular interactions between the planar binding surface of CBM and lignin (enzyme model: GH5 endoglucanase EglS, AlphaFold DB accession ID: AF-P10475-F1). See Supplementary Tables 4, 5 for relevant studies on creating multi-functional chimera and reducing non-productive binding to lignin, respectively.

#### 4.4.1 Designing multi-functional PCWPEs for improved synergy

Fusing multiple functional domains into a single polypeptide chain has been demonstrated to enhance interdomain synergy in cellulases and xylanases, establishing enzyme fusion as a promising strategy for degrading multiple cell wall substances (Brunecky et al., 2017; Chu et al., 2019; Kmezik et al., 2020). This approach leverages the modular architecture of certain PCWPEs, which typically combined CD, CBM and carbohydrate esterase domain (CE) with flexible linkers (Xu et al., 2018).

Currently, researchers have constructed multifunctional chimeras by integrating functional domains derived from the same or distinct microbial sources (Supplementary Table 4). Wang et al. (2022) developed a bifunctional enzyme, XynII-Fae, by fusing a GH11 β-xylanase (XynII) from *Trichoderma reesei* with a feruloyl esterase domain (Fae) from GH10 β-xylanase Xyn-Fae identified in *Prevotella ruminicola* 23. It increased xylan and glucan conversion by 125.1% and 54.3%, respectively, and boosted wheat straw/sugarcane bagasse hydrolysis when combined with commercial cellulase (Wang et al., 2022). On this basis, the same group engineered a triple-functional enzyme by fusing a β-glucosidase from *Thermobifida fusca* to Xyn-Fae via a flexible linker (GGGGS)_3_. The resulting triple-functional enzyme (GXF) exhibited significantly enhanced activities compared to its parents. Furthermore, GXF effectively improved the aroma and quality of Longjing tea (Wang et al., 2024), highlighting the potential of multi-functional PCWPEs in food processing. Similarly, to facilitate a cost- effective biomass degradation, Liang et al. (2025) fused a bi-functional xylanase/glucanase, Xyn (Glu)A from *Bacillus aerius* KW1 (isolated from Massa Medicata Fermentata) with its cognate unimodular feruloyl esterase, FaeB, using a flexible linker L1 (SAGSSAAGSGSG) to create a trifunctional chimera. This chimera outperformed the commercial cellulase LsCel in sugar yield from pretreated corn stover during an 8-h fermentation (Liang et al., 2025).

Collectively, these findings indicate that the strategic advantage of enzyme fusion resides in the rational, substrate-guided assembly of distinct catalytic and auxiliary functional modules. It necessitates a deeper understanding of the biochemical properties, structural features, and synergistic interplay between selected functional domains. For instance, the fusion of a GH5 endoglucanase with a GH48 cellobiohydrolase can achieve synergetic hydrolysis toward crystalline cellulose (Moraïs et al., 2012). Combining a GH10 β-xylanase with a GH48 CBH can act synergistically on both cellulose and xylan, as demonstrated in the bifunctional enzyme *Cb*Xyn10C/Cel48B created by fusing corresponding enzymes sourced from *Caldicellulosiruptor bescii* (Chu et al., 2019). Incorporating CBM can not only provide targeting and anchoring to specific glycans (García-Paz et al., 2024), but also swell the crystalline surface and expose more cleavage site for CDs (Reyes-Ortiz et al., 2013). CE module removes the barrier of polysaccharide backbones via deacylation, facilitating the hydrolysis of more complex lignocelluloses (Khalili Ghadikolaei et al., 2017). To support this, a functional module library (Figure 5A) should be built by analyzing sequences, structures, functions, arrangements, and known synergistic partnerships among CDs, CBMs, CEs, also rare domains like chitin-binding modules and fibronectins. Furthermore, lengths, compositions, and flexibilities of linkers should be integrated into this framework based on empirical evidence. Ultimately, this approach will guide the design of tailored enzyme chimeras for specific uses. Nevertheless, it should be noted that even though enzyme fusion enables the creation of bi- or even tri-functional chimeras, the degradation of branched polysaccharides still necessitates complex, multi-enzyme systems. Aishwarya and Goyal (2025) developed a tailored cocktail comprising a bifunctional cellulolytic chimera (EG-BGL fusion), a CBH, and a β-xylanase (all from *Clostridium thermocellum*); a β-xylosidase from *Bacillus ovatus* and an α-L-arabinofuranosidase from *Pseudopedobacter saltans* at a ratio of 9:4:2:2:3. This cocktail achieved a saccharification efficiency of 76% toward pretreated elephant grass (Aishwarya and Goyal, 2025). From this, integrating enzyme fusion with tailored formulation of synergistic enzyme cocktails would be a future avenue to achieve more efficient saccharification.

#### 4.4.2 Reducing non-productive binding to lignin

The non-productive binding of cellulases to lignin is a key limiting factor in the enzymatic saccharification of lignocellulosic biomass, primarily driven by hydrophobic and electrostatic interactions between the enzymes and lignin (Rahikainen et al., 2013). Gao et al. (2014) further identified that aromatic residues within CBMs are critical for this non-productive adsorption. Moreover, an alanine-scanning studies on the CBM of *Trichoderma reesei* Cel7A revealed that the planar, cellulose-binding face constitutes the primary interface mediating lignin affinity (Strobel et al., 2015). Although rational engineering strategies specifically aimed at reducing non-productive lignin binding remain limited (Supplementary Table 5), emerging progress indicates that modulating the surface charge of CD and CBM constitutes a promising avenue.

Whitehead et al. (2017) used Rosetta-guided design (Leaver-Fay et al., 2011) to add negative surface charge to the CelE catalytic domain (CelE-CD) and CBM3a from *Ruminoclostridium thermocellum*. Surface residues at least 10 Å from any active site were replaced with Asp or Glu. The designs suggested that electrostatic repulsion might reduce adverse lignin interactions, but the same repulsion also reduced cellulose conversion, indicating that excessive negative surface charge is not conducive to effective interaction with cellulose fibers (Whitehead et al., 2017). The same group designed a combinatorial library comprising 33 variants with systematically varied architectures of the *Thermobifida fusca*-derived CBM2a and Cel5A, covering a net charge range from −52 to +37. The mutant D3 CBM2a-WT Cel5A, with a charge of +6.5, showed higher activity on both cellulose and lignin-enriched corn stover than the wild-type enzyme (DeChellis et al., 2024).

Based on limited current research, engineering the CBM domain is a promising strategy to reduce non-productive lignin binding in lignocellulosic biomass (Figure 5B). However, it would be more effective to first quantify the binding differences of studied CBMs between cellulose and lignin, using methods like GFP-fusion (Li et al., 2018) and then select those with higher cellulose selectivity for further engineering. Regarding engineering strategies, in addition to the aforementioned surface supercharging to enhance electrostatic repulsion, weakening hydrophobic interactions on the planar, cellulose-binding surface of CBMs represents a complementary engineering approach (Strobel et al., 2015). This is exemplified by the comparison of Cel7A and Cel7B from *Trichoderma reesei*. The more hydrophobic planar surface of the CBM makes Cel7B exhibit higher affinity to lignin (Börjesson et al., 2007).

### 4.5 Alleviating product inhibition

Product inhibition in PCWPEs is a multifaceted phenomenon exhibiting competitive, non-competitive, or mixed kinetic behavior (Baksi et al., 2023). Current research efforts in this field primarily target cellulase systems, where accumulating oligo- and monosaccharides impair synergistic interactions among endoglucanases, exoglucanases, and β-glucosidases (Andrić et al., 2010). To mitigate this inhibition, protein engineering strategies typically focus on weakening product binding or facilitating product release.

To date, most engineering studies addressing product inhibition have focused on β-glucosidases (Supplementary Table 6). Blocking the glucose access or binding has been shown to effectively mitigate this inhibition. Kamale et al. (2025) modified the active crater of a GH1 β-glucosidase from *Acetivibrio thermocellus* (*At*GH1). By comparing structures and sequences with glucose-tolerant homologs Bgl6 and UnBgl1 from soil metagenomes, Gly168 and Ser242 were mutated into the bulkiest Trp residues to introduce steric hindrance at these positions. The resulting mutants G168W and S242W showed prominently higher glucose tolerance with *IC*_50_ values of 840 mM and 612 mM compared to 380 mM for the wild-type (Kamale et al., 2025). Structural analysis revealed that the bulky side chain in G168W created substantial steric hindrance, limiting glucose binding at the +1 subsite. The S242W mutation narrowed the entrance of the active-site crater (from a width of 20.5 Å to 16.8 Å), thereby impeding glucose access. A similar entrance-narrowing mechanism has been documented in the L167W/P172L double mutant of a GH1 β-glucosidase *Th*Bgl derived from *Trichoderma harzianum*, which also displays enhanced glucose tolerance (Santos et al., 2019). In a separate study on a β-xylosidase Xyl21 from saline soil, directed evolution yielded a variant Xyl21-A16 (K16R/L94I/K262N) with enhanced xylose tolerance (Zhang et al., 2022). It was tentatively assumed that the K16R and K262N substitutions strengthened hydrogen bonding with neighboring residues, thereby enhancing structural rigidity and improving tolerance to xylose inhibition.

Currently, the understanding of product inhibition mechanisms in PCWPEs remains limited. Hegazy’s study demonstrated that xylose bound to a distal, small pocket located between N-terminal α-helices 3 and 4 of a *Geobacillus stearothermophilus*-derived β-xylanase XT6, thereby inducing non-competitive inhibition (Hegazy et al., 2019). Through directed evolution, they identified two xylose-tolerant variants, L133V and M116I/L131P. Structural analysis indicated that the substitutions reduce the van der Waals volumes, which further enhanced the mobility of N-terminal α-helices 3 and 4, thereby attenuating the inhibitory impact of xylose binding on catalytic function. Additionally, a landmark 2024 study employing single-molecule tracking, elucidated a dual-mode inhibitory mechanism of *Trichoderma reesei* Cel7A. Specifically, cellobiose binding to the substrate-entry tunnel (front door) blocks the association of Cel7A with cellulose, while concurrent occupancy of the product-release site (back door) reduces processive velocity and travel distance per binding encounter (Nong et al., 2024).

Based on these findings, to alleviate product inhibition, engineering must target the specific inhibition mechanism. Figure 6 shows how kinetic analysis with the generalized inhibition equation identifies the underlying mechanism. Specifically, the values of α (defined as 1+[I]/*K*_*i*_) and α′ (defined as 1+[I]/*K_*i*_’*) determine whether inhibition is competitive, uncompetitive, or mixed (Zhi-Yi and Wong, 2005; Cornish-Bowden, 2012). Computational tools support further rational design: molecular docking (e.g., Autodock Vina, Discovery Studio) predicts energetically favorable inhibitor binding poses within canonical active sites or putative allosteric pockets (Lu et al., 2011; Hegazy et al., 2019); MD simulations further elucidate the dynamic conformational changes of the enzyme-inhibitor complex over time, identifying interfacial residues critical for binding stabilization. Notably, all *in silico* predictions require further experimental validation. Subsequently, site-directed mutagenesis enables precise perturbation of inhibitor binding either by introducing unfavorable interaction or steric hindrance that impedes inhibitor access.

**FIGURE 6:**
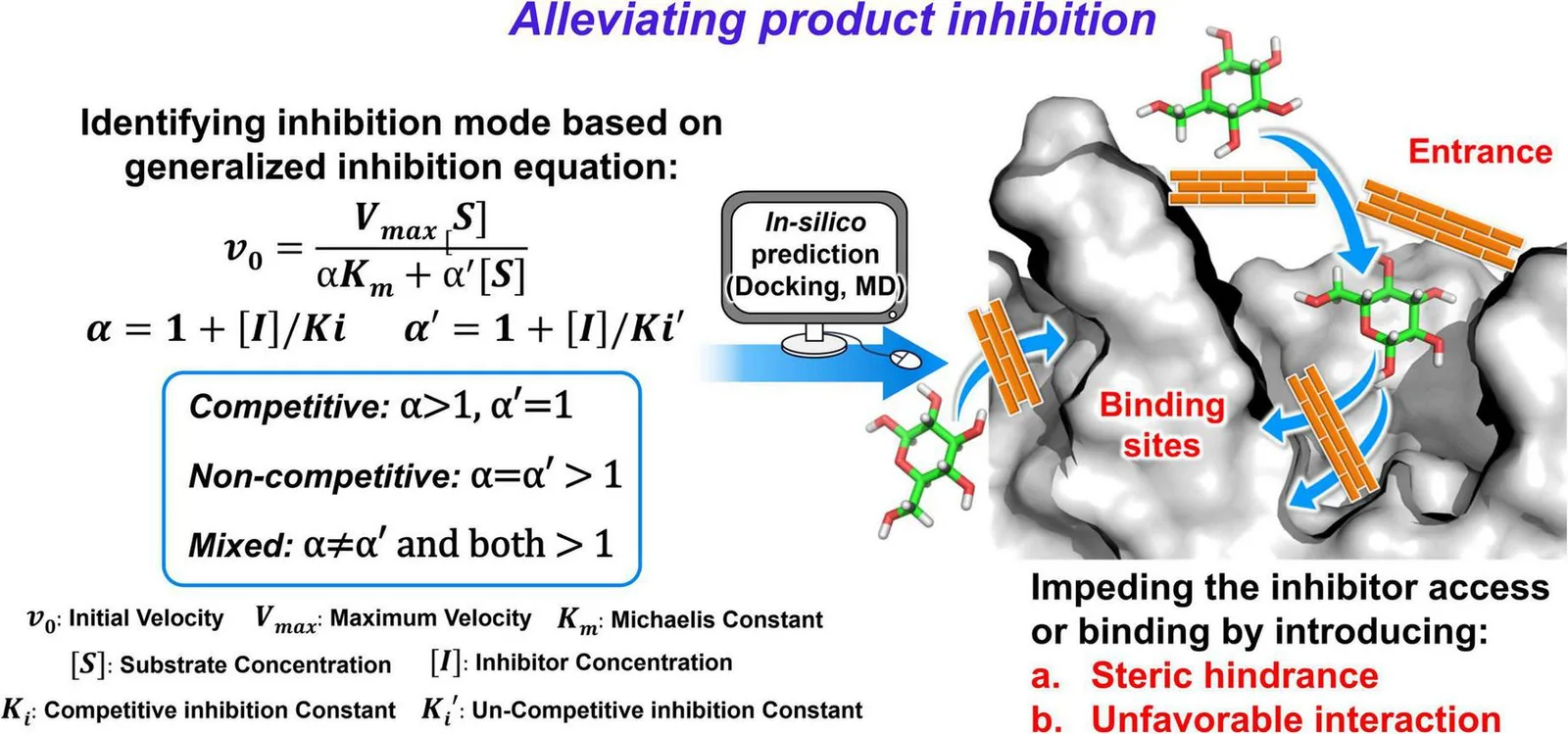
Proposed strategies for alleviating product inhibition should first identify the inhibition mechanism, then map the inhibitor-binding sites (e.g., glucose), and finally engineer targeted interventions to impede the access and subsequent binding of inhibitors. The enzyme model used here is GH11 β-glucosidase *At*GH1 (PDB ID: 5OGZ). See Supplementary Table 6 for relevant studies.

## 5 Future directions and recommendations

Enzyme engineering of microbial PCWPEs offers substantial promise for addressing persistent challenges in industrial biocatalysis. This review is designed to serve as a targeted, problem-driven reference: for each well-defined limitation encountered with a specific PCWPE, the evidence-based engineering strategies are mapped. Poor thermostability may be addressed by rigidifying flexible regions with high RMSF or B-factor. When nonproductive lignin binding limits activity, engineering can target the planar cellulose-binding face of the CBM and adjust intermolecular interactions. Nevertheless, based on the current body of literature, several critical bottlenecks remain unresolved in PCWPE engineering. Accordingly, future research priorities should focus on three frontiers outlined in Figure 7: First, to date, engineering efforts still focus on a narrow subset of PCWPEs, due to the following two main factors: (i) Researchers prioritize enzymes with high industrial potential and established markets, leaving other enzymes underexplored; (ii) Some model enzymes have accumulated a robust research foundation which lowers technical barriers to engineering. Accessory enzymes such as arabinofuranosidases, by contrast, still lack comprehensive structure-function insights, severely impeding their development (Sista Kameshwar and Qin, 2019). This focus reflects earlier technical constraints; the enzyme repertoire should now be broadened to include enzymes from a wider range of sources. Additionally, beyond conventional objectives such as catalytic efficiency and thermostability, greater emphasis should be placed on emerging priorities such as enhancing tolerance to high salinity and organic solvents, as well as mitigating non-productive enzyme adsorption onto lignin. These advances are essential to enable PCWPEs to function effectively in more complex application systems.

**FIGURE 7:**
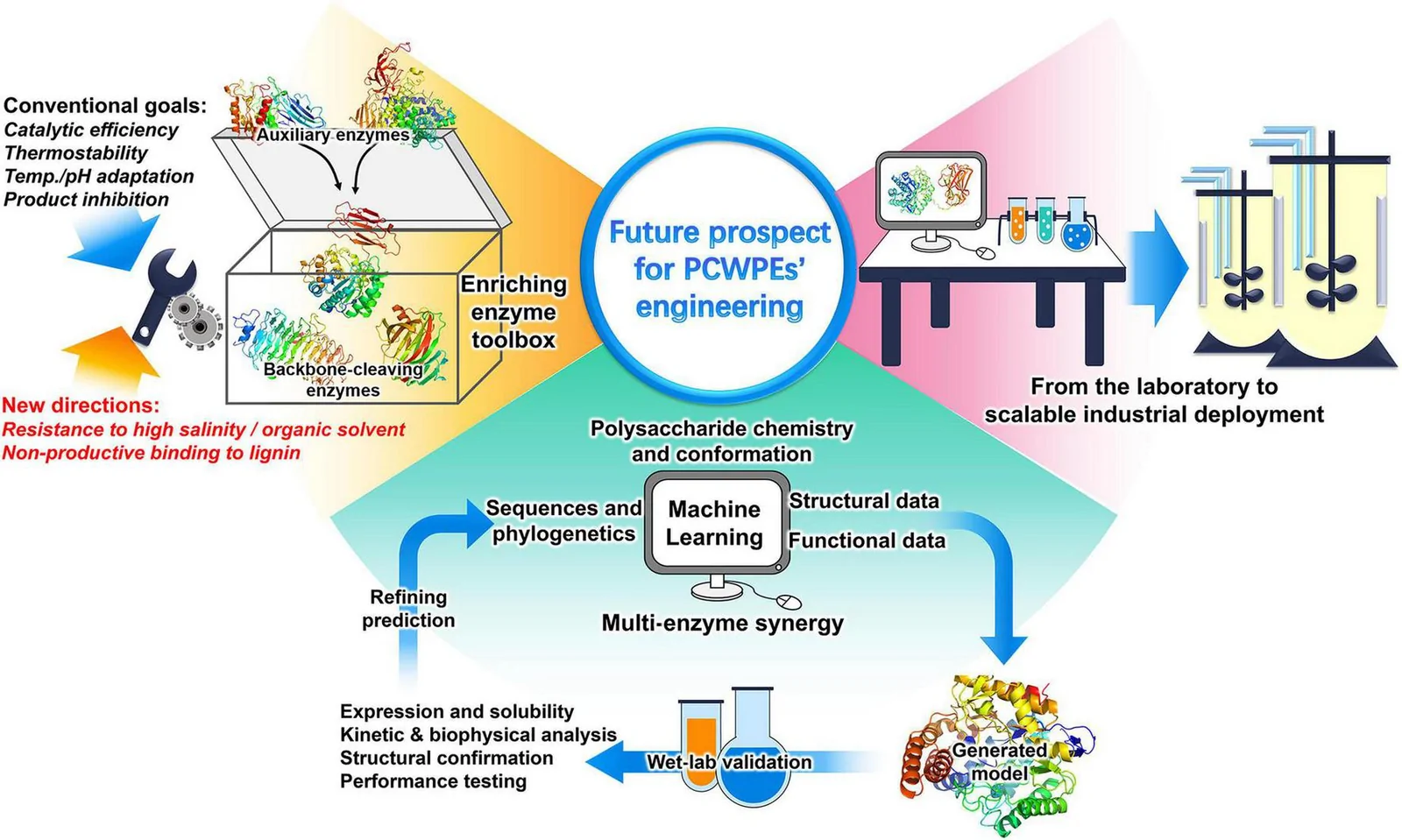
Future PCWPE engineering should focus on three priorities: (i) enriching PCWPE toolbox and further integrating more advanced engineering directions; (ii) Bridging the design-validation gap in machine learning to enable effective, feasible design; (iii) scaling promising variants from lab development to robust industrial deployment, unlocking their full potential for sustainable biomanufacturing. The enzyme models used here include GH5 endoglucanase EglS (AlphaFold DB accession ID: AF-P10475-F1), GH11 β-xylanase XynB (AlphaFold DB accession ID: AF-P17137-F1), GH127 β-L-arabinofuranosidase (PDB ID: 7EXW), pectate lyase Pel9A (AlphaFold DB accession ID: AF-E0RL41-F1), CE2 carbohydrate esterase (PDB ID: 4XVH).

Machine learning (ML) has seen limited use in PCWPE engineering. ML accelerates enzyme engineering by learning complex sequence-structure-function relationships (Siedhoff et al., 2020). It surpasses traditional approaches like directed evolution and rational design, which are often constrained by experimental burdens and insightful structural analysis (Singh et al., 2021). Generative AI tools (e.g., RFdiffusion, ProGen) even enable *de novo* design of functional enzymes like serine hydrolases (Lauko et al., 2025) and PET hydrolases (Rohan Ali, 2024). However, transforming promising ML designs into validated high-performance PCWPEs remains challenging because predictions still do not translate reliably into wet-lab results. This problem cannot be addressed without consistent generation, validation, and storage of machine-readable experimental data; without it even the most advanced models have limited practical utility (Markus et al., 2023; Zhou and Huang, 2024). Training data remain sparse in several areas, including (i) sequences and phylogenetics; (ii) the chemical and conformational diversity of polysaccharides; (iii) structural data on enzyme-substrate complexes and functional sites; (iv) systematic functional data (optima, stability, kinetics, substrate spectrum) better linked with structure (Krishnan et al., 2025) and (v) quantitative parameters describing multi-enzyme synergy (e.g., spatial proximity, substrate channeling efficiency). After model generation, variants must be validated experimentally through high-throughput characterization covering expression and solubility, kinetic and biophysical analysis, structural characterization and performance testing under application-relevant conditions (Vasina et al., 2022). Experimental results should then be incorporated into the data set into the ML training cycle through a closed-loop, iterative framework to refine predictions and progressively bridge the design-validation gap (Yang et al., 2019). As this framework matures with expanding datasets, industry-targeted PCWPE engineering will become increasingly effective and feasible. To date, Gado et al. (2021) pioneered ML-based functional classification of GH7 cellulases, training a model on 1,748 sequences to distinguish cellobiohydrolases from endoglucanases, identifying loop lengths (A4, B2, B3, B4) as key discriminative features. Building on this approach, integrating deep learning with high-quality experimental data will bridge the current data gap and enable ML-driven engineering of PCWPEs for high-performance industrial variants.

Last but not the least, to date, most engineering studies on PCWPEs remain confined to the laboratory, where performance is evaluated mainly through biochemical parameters measured on purified enzymes with synthetic substrates under ideal, non-competitive conditions. Such assays, while useful for fundamental exploration, often translate poorly to real production environments. Industrial operations are characterized instead by high solid loadings, elevated viscosity and mass-transfer limitations, the presence of inhibitors and by-products, and fluctuating variables such as temperature, pH, or stirring speed (Huang et al., 2022; Almeida et al., 2025; Hall et al., 2025). A recent perspective on protein engineering for industrial applications has explicitly noted that current explorations focus on scientific breakthroughs rather than market trends, commercialization strategies, or industry challenges (Gonlepa et al., 2025). Bridging this gap demands that engineered enzymes with improved functional properties move from laboratory development to robust, scalable industrial deployment. Concretely, this means adopting standardized metrics that reflect enzyme performance under industrially relevant conditions, prioritizing scale-up and pilot-plant validation of the most promising candidates, and strengthening collaboration between academic researchers and industry. Only by closing this divide can the full potential of PCWPEs be unleashed in biomanufacturing.

## Footnotes

1
http://dezyme.com/en/Software

2
https://loschmidt.chemi.muni.cz/fireprotasr/
