# Supplementary material for: Unleashing the industrial potential of microbial plant cell wall polysaccharide-degrading enzymes: recent advances in protein engineering
Source: Front Microbiol. 2026 Oct 13;17:1945309. doi: 10.3389/fmicb.2026.1945309 (PMC13710976; doi:10.3389/fmicb.2026.1945309)
Supplement: Supplementary file 1 [file Table_1.docx]

Supplementary Material

# 1 Supplementary Figures and Tables

## 1.1 Supplementary Tables

**Supplementary Table 1**. Summary of strategies on engineering PCWPEs to enhance catalytic efficiency. Representative studies published from 2015 onward were included, with preference given to those providing structural insights into the improvement.

| **Microbial sources** | **Engineering strategy** | **Improvement**  **(Activity)** | **Improvement**  **(*k_cat_/K_m_*)** | **Works** |
| --- | --- | --- | --- | --- |
| ***Bacillus subtilis*** | **Endoglucanase EG5C-1 (SRD):**  Employing alanine scanning to identify candidate sites flanking the cellohexaose-binding site, followed by saturation mutagenesis to optimize catalytic activities. | **Substrate:** CMC-Na  **WT:** 124.0 U/mg  **D70Q/S235W:** 261.0 U/mg | **Substrate:** CMC-Na  **WT:** 17.9 mL/mg/s  **D70Q/S235W:** 37.3 mL/mg/s | (Lv et al., 2021) |
| ***Bacillus subtilis*** | **Endoglucanase EG5C-1 (RD+SRD):**  Mutating the potential residues in catalytic domain guided by consensus analysis and optimizing five residues in loop region (G230-F241) involved in substrate binding by saturation mutagenesis. | **Not mentioned.** | **Substrate:** CMC-Na  **WT:** 12.1 mL/mg/s  **K91I/A198T/Q237D/V240P:** 103.2 mL/mg/s. | (Lv et al., 2024) |
| ***Chaetomium thermophilum***  **And**  ***Trichoderma reesei*** | **Cellobiohydrolase I (EF):**  Fusing the carbohydrate-binding module (CBM) of *Chaetomium thermophilum* CBHI with the catalytic domain (CD) and linker of *Trichoderma reesei* CBHI. | **Substrate:**  4-methylumbelliferyl-β-D-cellobiose  **WT:** 282.5 U/μM  **CT-CBH:** 734.5 U/μM | **Substrate:** PASC  **WT:** 3.**7** mL/mg/s  **CT-CBH:** 6.0 mL/mg/s. | (Xue et al., 2025) |
| ***Penicillium oxalicum* 16** | **β-glucosidase 16BGL (DE):**  Constructing mutant libraries by error-prone PCR and screening positive colonies via the chromogenic reaction of 6-(β-D-glucopyranosyloxy)-7-hydroxy-2H-1-benzopyran-2-one, followed by further activity confirmation using the DNS method. | **Not mentioned.** | **Substrate:** *p*-nitrophenyl-  β-D-glucopyranoside (*p*NPG)  **WT:** 807 mM^-1^ s^-1^  **Y-1-B1 (G414S/D421V/T441S):**  1355 mM^-1^ s^-1^ | (Huang et al., 2020) |
| ***Bacillus amyloliquefaciens*** | **LPMO *Ba*LPMO10A (RD):**  Mutating specific residues to more frequent ones based on consensus analysis with its homologues (identity between 50.0% and 66.1%). | **This work provided only relative activities.**  **Substrate:** 2,6-DMP  **WT:** 100.0%  **V40L/E124D:**193.7% | **No definite data. The estimated values were inferred from bar chart.**  **Substrate:** 2,6-DMP  **WT:** ~0.061 mM^-1^ min^-1^  **V40L/E124D:**  ~0.135 mM^-1^ min^-1^ | (Berhe et al., 2023) |
| ***Streptomyce rochei* L10904** | **β-Xylanase SrXyn (RD):**  Replacing the N-terminal “ATTITT” with β-strand A1 “AFTVGNGQ” of a xylanase from *Neocallimastix patriciarum* and mutating Ile99 to Thr in the cord, based on alignment with the homologue from *Streptomyces* sp. JHA19. | **Substrate:** Beechwood xylan  **WT:** 58.2 U/mg  **SrxynFM:** 310.0 U/mg | **Substrate:** Beechwood xylan  **WT:** 1.8 mL/mg/s  **SrxynFM:** 3.7 mL/mg/s | (Li et al., 2017a) |
| ***Bacillus subtilis*** | **β-Xylanase XynLC9 (SRD):**  Targeting N-terminal residues ^5^-YWQN-^8^ via sequence and structural comparisons with the Xyn2  from *Trichoderma reesei* and Xyn11A from *Thermobifida fusca*, followed by site saturation and iterative mutagenesis to optimize catalytic activities. | **Substrate:** Beechwood xylan  **WT:** 603.8 U/mg  **W6F/Q7H:** 1,507.1 U/mg | **Substrate:** Beechwood xylan  **WT: 7**.8 mL/mg/s  **W6F/Q7H:** 11.4 mL/mg/s. | (Wang et al., 2021) |
| ***Bacillus* sp. 30Y5** | **β-Xylanase** **Xyn30Y5 (RD):**  Designing mutants based on eight rational strategies like stabilizing specific regions, facilitating substrate accessing and product releasing, reducing steric hinderance of long-chain residues in substrate binding region. | **Substrate:** Beechwood xylan  **WT:** 349.4 U/mg  **Mutant 3B**  **(G229S/M225I/V245L/D293Q/D322N):** 1,016.8 U/mg | **Substrate:** Beechwood xylan  **WT:** 275.4 mL/mg/s  **Mutant 3B**  **(G229S/M225I/V245L/D293Q/D322N):** 547.2 mL/mg/s | (Lai et al., 2020) |
| ***Aspergillus kawachii***  **IFO 4308** | **β-Mannanase ManAK (RD):**  Replacing the Cys292, Cys293, and Leu294 near active site Glu296 based on the sequence and structure alignments with homologue ManCbs from *Cellulosimicrobium* sp. HY-13. | **Not mentioned.** | **Substrate:** D-mannan  **WT:** 199.3 mL/mg/s  **C292V:** 803.2 mL/mg/s | (Liang et al., 2021) |
| ***Bacillus* sp.**  **MK-2** | **β-Mannanase Bman26 (DE):**  Constructing mutant libraries by error-prone PCR and screening positive colonies by measuring diameters of halos on trypan blue solid medium plates. | **Substrate:** Konjac glucomannan  **WT:** 2,802 U/mg  **K291E:** 8,760 U/mg | **Substrate:** Konjac glucomannan  **WT:** 761.1 mL/mg/s  **K291E:** 3,456.6 mL/mg/s | (Zhang et al., 2019) |
| ***Paenibacillus cineris*** | **β-Mannanase *Pc*Man113 (RD):**  Reshaping the substrate binding cleft by mutating the residues around the substrate-binding sites, but lacking specific selection criteria. | **Substrate:** Konjac gum  **WT:** 68.2 U/mg  **F110E/N246Y:** 313.7 U/mg | **Substrate:** Mannopentaose  **WT:** 6.8 mM^-1^ s^-1^  **F110E/N246Y:** 37.4 L mM^-1^ s^-1^ | (Sun et al., 2022) |
| ***Ruminiclostridium thermocellum*** | **α-L-arabinofuranosidase** ***Ct*Ara*f*51 (SRD):**  Mutating the apolar residues Leu29, Leu319 and Ile357 close to the hydroxymethyl group of non-natural substrate *p*-nitrophenyl-β-D-galactofuranoside (*p*NPGal) via saturation mutagenesis. | **Not mentioned.** | **Substrate:** *p*NPGal  **WT:** 500 mM^-1^ s^-1^  **L29V:** 1,623 mM^-1^ s^-1^ | (Pavic et al., 2019) |
| ***Bacillus subtilis*** | **Pectate lyase PEL168 (Multiple methods):**  **RD:** Improving the local hydrophobicity by **V132F**;  **DE:** Constructing mutant libraries by error-prone PCR and screening by CTAB indicator plates **(K47E)**;  **SRD:** Optimizing the residues at site 47 by saturation mutagenesis based on **K47E/V132F (K47D/V132F)**.  **MSA:** Further **i**ntroducing **R272W** guided by evolutionary trace method. | **Substrate:** Polygalacturonic acid  **WT:** 353.6 U/mg  **K47D/V132F/R272W:**  1544.4 U/mg | **Substrate:** Polygalacturonic acid  **WT:** 513.4 mL/mg/s  **K47D/V132F/R272W:**  1567.7 mL/mg/s | (Wang et al., 2018c) |
| ***Penicillium* sp.** | **Polygalacturonase PG63 (RD):**  Mutating 38 candidate sites to Lys or Tyr prone favorable to enable cation-π interactions and further employing MD simulations to assess their occupancy rates. | **Substrate:** Polygalacturonic acid  **WT:** 1,600 U/mg  **H58Y:** 14,900 U/mg | **Substrate:** Polygalacturonic acid  **WT:** 2.6×10^2^ mL/mg/s  **H58Y:** 87.0×10^2^ mL/mg/s. | (Tu et al., 2016) |
| ***Verticilium dahliae*** | **Pectate lyase *Vd*PelB (RD):**  Mutating the residues possible involved in the substate recognition or catalytic mechanism of the enzyme. | **This work provided only relative activities.**  **Substrate:** Polygalacturonic acid  **WT:** ~72%  **G125R:** ~100% | **Substrate:** Polygalacturonic acid  **WT:** 15.0 mL/mg/s  **G125R:** 14.5 mL/mg/s. | (Safran et al., 2023) |

All enzyme engineering strategies are presented by abbreviations as follows: “DE” stands for directed evolution, “RD” for rational design, “SRD” for semi-rational design, “EF” for enzyme fusion, “ML” for machine learning and “MSA” for multiple sequence alignment. “WT” stands for the wild-type enzyme.

**Supplementary Table 2**. Summary of strategies on engineering PCWPEs to enhance thermostability. Representative studies published from 2015 onward were included, with preference given to those providing structural insights into the improvement.

| **Microbial sources** | **Engineering strategy** | **Improvement**  **(*T_m_* or *T_50_* or *T_ddt_*)** | **Improvement**  **(Thermal deactivation)** | **Works** |
| --- | --- | --- | --- | --- |
| ***Talaromyces emersonii***  **And**  ***Stegonsporium opalus*** | **Endoglucanase *So*Cel5A (EF):**  Swapping the individual (βα) modules within (βα)_8_-barrel scaffold with structurally homologous modules from the highly thermostable *Te*Egl5A. | ***T_m_*:**  **WT:** 53.6 ℃  **Hybrid H8:** 76.5 ℃  **Hybrid H9:** 70.1 ℃ | **Half-life (*t_1/2_*) at 55 ℃**  **WT:** 0.4 h  **Hybrid H8:** 260 h  **Hybrid H9:** 96 h | (Zheng et al., 2019) |
| ***Chaetomium thermophilum*** | **Endoglucanase *CT*endo45 (RD):**  Inactivating the N-glycosylation sequon (N88-E89-T90) and decreasing entropy by mutating Tyr173 to Phe. | ***T_m_*:**  **WT:** 56.3 ℃  **T90A/Y173F:** 83.4 ℃ | **Residual activity**  **(treatment at 80 °C for 300 min):**  **WT:** 20.2%  **T90A/Y173F:** 88.6% | (Han et al., 2020) |
| ***Trichoderma reesei*** | **Endoglucanase EGⅠ and EGⅡ (ML):**  Optimizing flexible loop regions by combining machine learning on 350,000 existing primary protein sequences and B-factor analysis. | **Not mentioned.** | **Residual activity**  **(treatment at 70 °C for 30 min):**  **EGⅠ-WT:** 24.6%  **EGI-N287I:** 87.5%  **EGⅡ-WT:** 23.7%  **EGI-N287I:** 77.8% | (Gao et al., 2023) |
| ***Hypocrea jecorina*** | **Cellobiohydrolase Cel7A (DE):**  Identifying the stabilizing mutations through random mutagenesis and then combining the beneficial substitutions to generate thermostable variants capable of withstanding 69 °C heat treatment. | ***T_m_*:**  **WT:** 62.5 ℃  **FCA398 (18 mutations):**  72.9 ℃ | **Half-life (*t_1/2_*) at 69 ℃**  **WT:** 8.6 min  **FCA398 (18 mutations):** 75.3 min | (Goedegebuur et al., 2017) |
| ***Holtiella tumoricola*** | **β-Glucosidase Ks5A7 (DE):**  Performing four rounds of random mutagenesis and positive colonies identified by incubation at 60 °C for 40 min followed by staining with 0.1 % (w/v) esculin and 0.25 % (w/v) ferric ammonium citrate. | ***T_50_^10^*:**  **WT:** 40.9 ℃  **4R1 (5 mutations):**  66.4 ℃ | **Half-life (*t_1/2_*) at 50 ℃**  **WT:** 1.0 min  **4R1 (5 mutations):** 8640.0 min | (Cao et al., 2018) |
| ***Bacillus* sp. D1** | **β-Glucosidase BglD2 (RD):**  Site-directed mutagenesis guided by amino acid preference analysis across 1,240 thermophilic enzymes:  (i) Substituting surface residues (D → E, N → K/R, W/Y → R) to enhance charge complementarity and promote formation of stabilizing salt bridges or hydrogen bonds;  (ii) Reinforcing hydrophobic core packing by internal substitutions (D/N → V, W/Y → L). | **Not mentioned.** | **Half-life (*t_1/2_*) at 40 ℃**  **WT:** 9.7 min  **D411E:** 50.5 min | (Qi et al., 2025) |
| ***Cellvibrio japonicus*** | **LPMO *Cj*LPMO10A (RD):**  Introducing disulfide bonds between Asn78-His116 and Asp59-Arg121) locating on loops with high B-factor. | ***T_50_^15^*:**  **WT:** 63.8 ℃  **N78C/H116C:** 67.3 ℃  ***T_m_*:**  **WT:** 54.6 ℃  **N78C/H116C:** 61.7 ℃ | **Half-life (*t_1/2_*) at 60 ℃**  **WT:** 47.1 min  **N78C/H116C:** 145.9 min | (Zhou et al., 2022) |
| ***Neocallimastix patriciarum*** | **β-Xylanase XynCDBFV (RD):**  Replacing the C-terminal ^207^-NGGA-^210^ with ^207^-SSGS-^210^ based on the normalized B-factor analysis and alignment with thermophilic bacteria xylanase NFX. | **Not mentioned.** | **Residual activity**  **(treatment at 80 °C for 1 h):**  **WT:** 47%  **Xyn-MUT (N207S/G208S/A210S):** 61% | (Han et al., 2017) |
| ***Streptomyces rameus* L2001** | **β-Xylanase XynA (RD):**  Replacing the N-terminal ^11^-TDNGFY-^16^ with ^11^-YHDGYF-^16^ based on alignment with thermophilic xylanase TfxA from *Thermomonospora fusca*. | **Not mentioned.** | **Half-life (*t_1/2_*) at 80 ℃**  **WT:** 56.6 s  **XynAR (T11Y/D12H/N13D/F15Y/Y16F):** 154.0 min | (Wu et al., 2023b) |
| ***Streptomyces rameus* L2001** | **β-Xylanase XynA (RD):**  Rigidifying the N-terminal flexible loop regions based on the MD simulations and conserved sequence analysis with 8 xylanases exhibiting excellent hydrolytic properties and 21 thermotolerant xylanases. | **Not mentioned.** | **Residual activity**  **(treatment at 90 °C for 12 h):**  **WT:** 0.0%  **Mut1:** 87.0%  **Mut2:** 97.0% | (Wu et al., 2023a) |
| ***Paecilomyces thermophila*** | **β-Xylanase XynM2 (RD):**  Designing beneficial mutations by combining sequence conservation analysis and ΔΔ*G*_fold_ calculation using three computational tools: Rosetta Cartesian_ddg, Rosetta ddg_monomer and FoldX. | ***T_m_*:**  **WT:** 82.8 ℃  **XynM9 (7 mutations):**  93.1 ℃ | **Half-life (*t_1/2_*) at 85 ℃**  **WT:** 32 min  **XynM9 (7 mutations):** 1150 min | (Lu et al., 2024) |
| ***Aspergillus saccharolyticus***  **JOP 1030-1** | **β-Xylanase XynASP (RD):**  Stabilizing a flexible cord region (^117^YGEYNPGSAGT^127^) by three strategies:  (i) Consensus analysis with 53 GH11  xylanases (identity between 30.0% and 60.0%);  (ii) Virtual saturation mutagenesis evaluating ΔΔG values;  (iii) Disulfide bond engineering guided by the prediction of DbD2 software. | ***T_m_*:**  **DSM4** *versus* **WT:**  No definite data. The observed improvement was described only as a 20 °C increase in *T_m._* | **Half-life (*t_1/2_*) at 50 ℃**  **WT:** 5.3 min  **DSM4 (T41W/E119C/G126C/ A144C):** 693.7 min | (Li et al., 2025) |
| ***Bacillus subtilis***  **TJ-102** | **β-Mannanase ManTJ102 (RD):**  Virtually introducing prolines to flexible regions 300-310 and 330-340 identified from MD simulations and B-factor analysis and then experimentally validating Ala336Pro substitution with the lowest mutation energy. | ***T_dtt_***  **(dynamics transition temperature):**  **WT:** 60 ℃  **Mutant336 (A336P):** 70 ℃ | **Half-life (*t_1/2_*) at 60 ℃**  **WT:** 5 min  **Mutant336 (A336P):** 120 min | (Wang et al., 2018b) |
| ***Aspergillus niger*** **CBS513.88** | **β-Mannanase *An*man (RD):**  Rigidifying surface-exposed loop regions exhibiting elevated B-factors by performing virtual saturation mutagenesis in Discovery Studio 2019, followed by screening for single mutations with prominently reduced ΔΔG values. | ***T_m_*:**  **WT:** 75.7 ℃  **Mut5 (E15C/S65P/A84P/A195P/T298P):** 77.7 ℃ | **Half-life (*t_1/2_*) at 70 ℃**  **WT:** 8.3 min  **Mut5 (E15C/S65P/A84P/A195P/T298P):** 73.4 min | (Tan et al., 2023) |
| ***Paenibacillus* sp. 0602** | **Pectate lyase PelN (RD):**  Mutating the unfavorable Gly and Pro residues located within the α-helix structures to Ala or Val, followed by screening based on mutation energy calculation in Discovery Studio 4.1. | ***T_m_*:**  **K93I/G241A** *versus* **WT:**  No definite data. The observed improvement was described only as a 1.6 °C increase in *T_m._* | **Half-life (*t_1/2_*) at 60 ℃**  **WT:** 33.5 min  **K93I/G241A:** 49.3 min | (Zhou and Wang, 2021) |
| ***Bacillus clausii* S10** | **Pectate lyase *Bac*PelA (SRD):**  Identifying potential residues located in unstable regions (loops or turns) by combinatorial analysis of B-FITTER (average B-factor) and HoTMuSiC  Algorithm (positive *ΔT_m_* values), followed by saturation mutagenesis at these potential sites. | ***T_50_^15^* (pH 10.0):**  **WT:** 59.0 ℃  **R150G/R216H/A238C: 67.5** ℃  ***T_50_^15^* (pH 10.0, 0.1 mM Ca^2+^):**  **WT:** 59.0 ℃  **R150G/R216H/A238C: 67.5** ℃ | **Residual activity**  **(treatment at 60 °C, pH 10.0 for 4 h):**  **WT:** 3.4%  **R150G/R216H/A238C:** 86.2% | (Zhou et al., 2023) |
| ***Bacillus licheniformis*** | **Pectate lyase PMGL-Ba (SRD+ML):**  Employing ML model to iteratively explore the combinatorial sequence space and construct highly thermostable higher-order mutants, building upon a foundation of 18 thermostable single-point variants identified via semi-rational design. | **Not mentioned.** | **Half-life (*t_1/2_*) at 75 ℃**  **WT:** 12.4 min  **P36 (9 mutations):** 836.4 min | (Zhang et al., 2024) |

All enzyme engineering strategies are presented by abbreviations as follows: “DE” stands for directed evolution, “RD” for rational design, “SRD” for semi-rational design, “EF” for enzyme fusion, “ML” for machine learning and “MSA” for multiple sequence alignment.

**Supplementary Table 3**. Summary of strategies on engineering PCWPEs to optimize adaptability to non-neutral pH. Representative studies published from 2015 onward were included, with preference given to those providing structural insights into the improvement.

| **Microbial sources** | **Engineering strategy** | **Improvement**  **(pH-activity)** | **Improvement**  **(pH-stability)** | **Works** |
| --- | --- | --- | --- | --- |
| ***Talaromyces leycettanus***  **JCM12802** | **β-Glucosidase Bgl3A (RD):**  Removing the potential *O*-glycosylation site by:  (i) substituting to corresponding sequence from a close homologue *Nf*BGL1 (73 % identity) with less *O*-glycosylation sites **(M1)**;  (ii) substituting potential *O*-glycosylation sites (Thr) by Ala **(M2)**. | **Mutant M1 and M2** showed broader operational pH range of 3.0 to 10.0, compared with the narrow range of 4.0–5.0 observed for the wild-type. | **Mutant M1 and M2** retained over 70% residual activity after 12 h of incubation at pH 5.0 and 50 °C, whereas the wild-type was fully inactivated under identical conditions. | (Xia et al., 2016) |
| ***Aspergillus niger* nl-1r** | **β-Xylanase** **XynB (RD):**  Mutating the Gln164 and Asp177 around the thumb loop regions based on the sequence alignment with nine alkalophilic homologues. | **XynB-177 (D177N/Q164K)** exhibited a shift in pH optimum from 5.0 to 5.5. | **XynB-177 (D177N/Q164K)** exhibited significantly enhanced alkaline stability relative to the wild-type after incubation under pH 10.0, 4 °C for 12 h. | (Fei et al., 2015) |
| ***Bacillus pumilus***  **HBP8** | **β-Xylanase** **XynHBN188A (DE):**  Constructing mutant libraries by error-prone PCR and screening positive colonies by observing the hydrolysis halos on LB agar plate supplemented with 0.5% (w/v) remazol brilliant blue-xylan. | **XynHBN188A217 (N42D/T149S)** exhibited approximately a 10% increase in enzymatic activity within the pH range of 6.5–7.0. | **XynHBN188A217 (****N42D/T149S)** retained over 70% residual activity after 12-h incubation across the pH range of 5.0–8.5 at 4 °C, whereas the wild-type retained less than 40%. | (Xiang et al., 2019) |
| ***Rhizomucor miehei*** | **β-Mannanase** ***Rm*Man5A (DE):**  Constructing mutant libraries by error-prone PCR and screening positive colonies on pH 5.0 congo red plate supplemented with 0.5% (w/v) locust bean gum (LBG). | **m*Rm*Man5A (Y233H/K264M N343S)** exhibited over 3-fold higher activity towards LBG, konjac powder and guar gum under pH 4.5, 65 °C; concurrently, its pH optimum shifted from 7.0 to 4.5. | **No notable advantage.** | (Li et al., 2017b) |
| ***Aspergillus niger*** | **Pectin lyase** **CpelA (RD):**  Altering the surface alkaline residues to acidic ones (Asp and Glu) through the guidance of supercharge program in ROSETTA 2020.50, followed by further validation using Rosetta Cartesian_ddg scan to exclude instable variants. | **M6 (S514D/S538E)** revealed 2.78-fold, 3.0-fold, and 3.11-fold higher activity than the wild-type enzyme at pH 3.0, pH 4.0, and pH 5.0, respectively. | **M6 (S514D/S538E)** retained over 90% residual activity following 2- h incubation at pH 3.0 and 40 °C, whereas the CpelA retained less than 50%. | (Li et al., 2024) |

All enzyme engineering strategies are presented by abbreviations as follows: “DE” stands for directed evolution, “RD” for rational design.

**Supplementary Table 4**. Summary of strategies on engineering PCWPEs to design multi-functional chimeras for better synergy. Representative studies published from 2015 onward were included.

| **Microbial sources** | **Engineering strategy** | **Improvement on catalyzing model substrate**  **(activity or *k_cat_/K_m_*)** | **Improvement on treating biomass** | | **Works** |
| --- | --- | --- | --- | --- | --- |
|  |  |  | **Reaction condition** | **Activity or conversion**  **or reducing sugar yield** |  |
| **Camel rumen metagenome** | **β-xylanase/CMB/cellulase (EF):**  Fusing the catalytic domain and CBM of xylanase XylC to a single-domain endoglucanase CelC to construct **Xyn-CBM-Cel.** | ***k_cat_/K_m_***  **Substrate1:** CMC-Na  **XylC:** N.d.  **CelC:** 34.0 mL/mg/s  **Xyn-CBM-Cel:**  285.6 mL/mg/s  **Substrate2:**  oat spelt xylan (OSX)  **XylC:**  **CelC:** 34.0 mL/mg/s  **Xyn-CBM-Cel:**  37.3 mL/mg/s | **Pretreated**  **Biomass1:**  Rice straw  **Biomass2:**  Barley straw  **Enzyme loading:**  Not mentioned.  **Conditions:**  Reacting under 35 °C, pH 5.0 to treat 20 mg/mL of biomass over a 2-h period. | **Activity**  **Biomass1:** Rice straw  **XylC:** 197.0 U/μmol  **CelC:** 8.4 U/μmol  **Xyn-CBM-Cel:**  398.4 U/μmol  **Biomass2:** Barley straw  **XylC:** 197.0 U/μmol  **CelC:** 8.4 U/μmol  **Xyn-CBM-Cel:**  398.4 U/μmol | (Khalili Ghadikolaei et al., 2017) |
| ***Caldicellulosiruptor saccharolyticus*** | **Endo-/exoglucanase (EF):**  Fusing endoglucanase (Endo5), exoglucanase (Exo5) with their cognate CBMs via a natural proline and threonine-rich linker (PT-box) to construct **Endo5-2CBM-Exo5** and **Endo5-CBM3b-Exo5.** | **Activity**  **Substrate:**  Crystalline cellulose  **Exo5:** 0.053 U/mg  **Endo5:** 0.011 U/mg  **Endo5-2CBM-Exo5:**  0.167 U/mg  **Endo5-CBM3b-Exo5:**  0.157 U/mg | **Un-pretreated**  **Biomass1:**  Rice straw  **Biomass2:**  Wheat straw  **Enzyme loading:**  10 μg of diluted purified enzymes.  **Conditions:**  Reacting under 80 °C, pH 4.5, with agitation at 200 rpm to treat 25 mg/mL of biomass over a 4-h period. | **Activity**  **Biomass1:** Rice straw  **Exo5:** untested  **Endo5:** untested  **Endo5-2CBM-Exo5:**  0.102 U/mg  **Endo5-CBM3b-Exo5:**  0.106 U/μmol  **Biomass2:** Wheat straw  **Exo5:** untested  **Endo5:** untested  **Endo5-2CBM-Exo5:**  0.043 U/mg  **Endo5-CBM3b-Exo5:**  0.078 U/mg | (Li et al., 2019) |
| ***Trichoderma reesei***  **And**  ***Prevotella ruminicola* 23** | **β-xylanase/feruloyl esterase (EF):**  Fusing β-xylanase from *Trichoderma reesei* with the C-terminal feruloyl esterase domain of Xyn-Fae from *Prevotella ruminicola* 23 to form **XynII-Fae**. | **Activity**  **Substrate:**  Beechwood xylan  **XynII-Fae:** 78.5 U/mg | **Pretreated**  **Biomass:**  Delignified corn stover  **Enzyme loading:**  6 ‰ (w/w) commercial cellulase  4 ‰ (w/w) XynII-Fae  **Conditions:**  Reacting under 40 °C, pH 6.0, with agitation at 200 rpm for to treat 2 % (w/w) of corn stover over a 120-h period. | **Reducing sugar yield:**  **Commercial cellulase:**  4.92 mg/mL  **XynII-Fae:**  1.77 mg/mL  **Commercial cellulase: XynII-Fae=6:4:**  9.47 mg/mL | (Wang et al., 2022) |
| ***Thermobifida fusca***  **And**  ***Prevotella ruminicola* 23** | **β-glucosidase/β-xylanase/**  **feruloyl esterase (EF):**  Fusing a β-glucosidase from *Thermobifida fusca* with a bifunctional β-xylanase /feruloyl esterase identified in Wang’s work (2022) via a flexible linker (GGGGS)_3_ to construct **multifunctional GFX**. | ***k_cat_/K_m_***  **Substrate1:** *p*NPG  **β-Glucosidase:**  149.8 mL/mg/s  **GFX:** 250.4 mL/mg/s  **Substrate2:**  Beechwood xylan  **β-Xylanase:**  40.7 mL/mg/s  **GFX:** 60.5 mL/mg/s  **Substrate3:**  *p*-nirtophenyl acetate  **Feruloyl esterase:**  63.9 mL/mg/s  **GFX:** 78.9 mL/mg/s  **Substrate4:**  Methyl ferulate  **Feruloyl esterase:**  89.7 mL/mg/s  **GFX:** 124.6 mL/mg/s | **Un-pretreated**  **Biomass:**  Longjing tea  **Enzyme loading:**  5 ‰ (w/w) for each.  **Conditions:**  Reacting in water under 40 °C, with agitation at 150 rpm to treat 10 % (w/w) of tea sample over a 120-h period. | **Conversion rate:**  **β-Glucosidase:**  1.78%  **β-Xylanase:**  1.42%  **Feruloyl esterase:**  1.37%  **β-Xylanase/feruloyl esterase:**  3.06%  **β-Glucosidase+β-xylanase + feruloyl esterase:**  4.19%  **β-Glucosidase+**  **β-xylanase/feruloyl esterase:**  4.71%  **GFX:** 5.26% | (Wang et al., 2024) |
| ***Bacillus aerius***  **KW1** | **β-xylanase/glucanase/ feruloyl esterase (EF):**  Fusing the bifunctional xylanase/glucanase Xyn (Glu)A from *Bacillus* with its cognate feruloyl esterase FaeB using an optimized flexible coil linker L1 (SAGSSAAGSGSG) to construct **XF-L1**. | ***k_cat_/K_m_***  **Substrate1:**  Wheat arabino xylan  **Xyn(Glu)A:**  181.31 mL/mg/s.  **FaeB:** N.d.  **XF-L1:**  268.5 mL/mg/s  **Substrate2:**  Barley β-glucan  **Xyn(Glu)A:**  0.6 mL/mg/s.  **FaeB:** N.d.  **XF-L1:**  5.6 mL/mg/s  **Substrate3:**  *p-*nitrophenyl ferulate  **Xyn(Glu)A:** N.d.  **FaeB:** 0.2 mM^-1^ s^-1^  **XF-L1:**  0.73 mM^-1^ s^-1^ | **Pretreated**  **Biomass:**  Corn stover  **Enzyme loading:**  0.5 μM for each.  **Conditions:**  Reacting under 50 °C, pH 6.0, with agitation at 700 rpm to treat 20 mg/mL of corn stover over an 8- h period. | **Reducing sugar yield:**  **FaeB:**  0.84 mM  **Commercial LsCel:**  4.27 mM  **FaeB+LsCel**  7.33 mM  **Xyn(Glu)A + LsCel:**  8.35 mM  **XF-L1 + LsCel:**  10.33 mM | (Liang et al., 2025) |
| ***Streptomyces thermoviolaceus***  **And**  ***Bifidobacterium adolescentis*** | **α-L-arabinofuranosidases (EF):**  Fusing a GH62 α-L-arabinofuranosidase (*Sth*Abf62A) catalyzing the removal of mono-substituted α-L-arabinofuranosyl residues (AXH-m) with a GH43 enzyme (*Bad*Abf43A) catalyzing the removal of double-substituted residues (AXH-d). | **Activity (× 10^3^)**  **Substrate1:**  Wheat arabinoxylan **SthAbf62A:**  320.4 U/mmol  **BadAbf43A:**  538.3 U/mmol  **SthAbf62A+BadAbf43A:**  860.3 U/mmol  **SthAbf62A_BadAbf43A:**  1117.9 U/mmol  **BadAbf43A_SthAbf62A:**  1240.5 U/mmol  **Substrate2:**  Di-2,3–arabinoxylan **SthAbf62A:**  N.d.  **BadAbf43A:**  727.3 U/mmol  **SthAbf62A+BadAbf43A:**  898.1 U/mmol  **SthAbf62A_BadAbf43A:**  1467.8 U/mmol  **BadAbf43A_SthAbf62A:**  1531.9 U/mmol | **Not mentioned.** | | (Wang et al., 2018a) |

“EF” for enzyme fusion. “N.d.” stands for not detectable.

**Supplementary Table 5**. Summary of strategies on engineering PCWPEs to reduce non-productive binding to lignin. Representative studies published from 2015 onward were included.

| **Microbial sources** | **Engineering strategy** | **Reaction condition** | **Improvement** | **Works** |
| --- | --- | --- | --- | --- |
| ***Ruminoclostridium thermocellum*** | **CelE-CD and CBM3a (RD):** Negatively supercharging the surfaces of CBM3a and CelE by substituting candidate positions located at least 10 Å away from active or substrate binding sites with Asp and Glu using Rosetta macromolecular software suite. | **Model substrate:**  PASC  **Enzyme loading:**  5 mg/g cellulose.  **Conditions:**  Reacting under 60 °C in 10 mM MOPS buffer to treat 4 mg/mL PASC in the absence or presence of lignin slurry (2 mg/mL) over a period of 120 h. | **Four mutants (D2-CD,** **D1-CBM3a, D2-CBM3a, D3-CBM3a)** with charges ranging from -16 to -25 showed enhanced lignin tolerance, but also a trade-off in cellulase activity.  Notably, **D3-CBM3a** exhibited higher PASC conversion in the presence of lignin, though lacking in-depth interpretation. | (Whitehead et al., 2017) |
| ***Thermobifida fusca*** | **Cel5A and CBM2a (RD):** Constructing a combinatorial library of 33 mutants featuring diverse supercharged CBM2a-Cel5A fusion architectures with net surface charges spanning -52 to 37. | **Pretreated**  **Biomass:**  Corn stover  **Enzyme loading:**  120 nmol/g biomass.  **Conditions:**  Reacting under 60 °C, pH 4.5-7.0, with agitation at 5 rpm to treat 2 mg of corn stover over a 24 h period. | **Mutant D3 CBM2a-WT Cel5A**, bearing a CBM with a net charge of +6.5, yielded more than twofold higher glucose relative the wild-type Cel5A at both pH 6.5 and pH 7.0. | (DeChellis et al., 2024) |

“RD” for rational design,

**Supplementary Table 6**. Summary of strategies on engineering PCWPEs to alleviate product inhibition. Representative studies published from 2015 onward were included, with preference given to those providing structural insights into the improvement.

| **Microbial sources** | **Engineering strategy** | **Inhibition**  **mode focused in the study** | **Inhibitor used in the test and**  **concentration** | ***IC_50_* or *K_i_*** | **Improvement**  **and trade off** | **Works** |
| --- | --- | --- | --- | --- | --- | --- |
| ***Rasamsonia emersonii*** | **Cellobiohydrolase *Te*Cel7A (RD):**  Mutating the residues within the cellobiose-binding site of Cel7A, which was expected to reduce the binding energy of cellobiose. | Competitive  inhibition | Thiocellobiose  4.39 g/L | The inhibitory potency of cellobiose was not quantified in terms of *IC_50_* or *K_i_*. Only dissociation constant (*K_d_*) of cellobiose was detected:  ***Te*Cel7A:** 17.9 μM  **Y385A:** 95.0 μM | **Y385A** exhibited a 19% increase in cellobiose tolerance relative to the wild-type, as evidenced by enhanced sugar release during **Avicel** hydrolysis.  **Trade-off:** a 17% reduction in activity under uninhibited conditions. | (Atreya et al., 2016) |
| ***Trichoderma harzianum*** | **β-Glucosidase *Th*Bgl (RD):**  Substituting Leu157 and Pro172 serving as gatekeepers of catalytic pocket based on the structural comparation with a glucose-tolerant homologue *Hi*Bgl (PDB ID: 4MDO). | Competitive  inhibition | Glucose  0.0 to 1.0 M | ***IC_50_*:** ~300 mM  No definite data. The estimated value was inferred by curve-fitting plot in the previous work of the same group (Santos et al., 2016). | ***Th*Bgl-Mut (L167W/P172L)** exhibited the highest catalytic activity across the glucose concentration range of 0.1–0.25 M, whereas the wild-type displayed a rapid decline in activity under identical conditions.  ***Th*Bgl-Mut** also enhanced glucose release by approximately 300% during the hydrolysis of **sugarcane material**.  **Trade-off:** None. | (Santos et al., 2019) |
| ***Acetivibrio thermocellus*** | **β-Glucosidase *At*GH1 (RD):**  Modifying the active site crater based on the sequence and structural comparison with three glucose-tolerant BGLs with high ***IC_50_*** values:  **(i) Bgl6** (*IC_50_*: 3.5 M);  **(ii) M3 mutant of Bgl6** (*IC_50_*: 3.0 M);  **(iii) C188V mutant of UnBGl1** (*IC_50_*:1.8 M). | Competitive  inhibition | Glucose  10 mM to 2.0 M | ***IC_50_*:** 380 mM | **G168W and S242W** exhibited improved glucose tolerance, with *IC_50_* values of 840 and 612 mM, respectively.  **Trade-off: G168W** showed prominently lower *k_cat_/K_m_* towards cellobiose (5.8 mM^-1^ min^-1^) relative to the wild-type (10.5 mM^-1^ min^-1^). | (Kamale et al., 2025) |
| **Microbial community of Amazonian soil** | **β-Glucosidase AMBGL18 (RD):**  Replacing the main residues contact with glucose and cellobiose, and identifying optimal residues for mutagenesis by pairwise alignment and ΔΔG prediction via *in-silico* thermostability analysis. | Competitive  inhibition | Glucose  500 mM | No quantitative data. | **W404L** possessed 3.2-fold increase in catalytic activity under 500 mM glucose compared to the wild-type.  **Trade-off:** None. | (Azevedo et al., 2025) |
| ***Bacillus subtilis***  **And**  ***Escherichia coli*** | **Xylose binding protein/β-xylanase (EF):**  Randomly linearizing the XBP (*E. coli*) encoding sequence with DNase Ⅰ and ligated with the XynA (*B. subtilis*) coding sequence to create a random XynA-XBP insertion library. | Cannot be judged due to the absence of structural interpretation on enzyme-inhibitor complex. | D-Xylose  1.0% (w/v) | No quantitative data. | **XynA-XBP271** exhibited a 3-fold increase in catalytic efficiency in hydrolyzing Remazol Brilliant Blue xylan (RBB-xylan) relative to the parental xylanase under 1 % (w/v) D-xylose.  **XynA-XBP271** also exhibited 37% increase in sugar release during the hydrolysis of **sorghum stover**.  **Trade-off:** None. | (Ribeiro et al., 2016) |
| ***Geobacillus stearothermophilus*** | **β-Xylanase XT6 (DE):**  Constructing mutant libraries by error-prone PCR, and screening positive colonies by catalyzing RBB-xylan under pH 7.6, 40 ℃ in the absence or the presence of 25 mM D-xylose. | Non-competitive  inhibition | D-Xylose  0 to 100 mM | ***K_i_*:** 12.2 mM | **L133V** showed enhance xylose tolerance, indicating by a 3.4-fold higher ***K_i_* (41.2 mM)** relative to the wild-type.  **M116I/L131P** also showed a 2.5-fold higher ***K_i_* (31.1 mM)** towards xylose.  **Trade-off:** None. | (Hegazy et al., 2019) |
| **Soil environment DNA** | **β-Xylosidase Xyl21 (DE):**  Constructing mutant libraries by error-prone PCR and screening positive colonies by detecting the activity within a 20-min reaction towards 4-nitrophenyl-D-xylopyranoside at 45 ℃ in the presence of 0.9 M xylose. | Cannot be judged due to the absence of structural interpretation on enzyme-inhibitor complex. | D-Xylose  0.3 to 2.1 M | No quantitative data. | **Xyl21-A16 (K16R/L94I/K262N)** retained higher residual activity (24.82–87.55%) across the xylose concentration range of 0.6–2.1 M, where the wild-type exhibited 21.48–74.31% residual activity.  **Trade-off:** Cannot be judged due to the absence of activity or kinetic test without xylose. | (Zhang et al., 2022) |

All enzyme engineering strategies are presented by abbreviations as follows: “DE” stands for directed evolution, “RD” for rational design, “SRD” for semi-rational design, “EF” for enzyme fusion. “Trade-off” denotes the reduction in catalytic capability observed in the absence of inhibitor following protein engineering compared to the wild-type. If there is no reduction in activity or *k_cat_/K_m_*, it is marked as “None”.

# 2 References

Atreya, M.E., Strobel, K.L., and Clark, D.S. (2016). Alleviating product inhibition in cellulase enzyme Cel7A. *Biotechnology and Bioengineering* 113(2)**,** 330-338. doi: 10.1002/bit.25809.

Azevedo, R.D.S., Santana, H., Seus, V.R., Camargo, A.D., Werhli, A.V., Machado, K.D.S., et al. (2025). Development of a beta-glucosidase improved for glucose retroinhibition for cellulosic ethanol production: an integrated bioinformatics and genetic engineering approach. *Biotechnology for Biofuels and Bioproducts* 18(1)**,** 44. doi: 10.1186/s13068-025-02643-4.

Berhe, M.H., Song, X., and Yao, L. (2023). Improving the Enzymatic Activity and Stability of a Lytic Polysaccharide Monooxygenase. *International Journal of Molecular Sciences* 24(10)**,** 8963. doi: 10.3390/ijms24108963.

Cao, L., Li, S., Huang, X., Qin, Z., Kong, W., Xie, W., et al. (2018). Enhancing the Thermostability of Highly Active and Glucose-Tolerant β-Glucosidase Ks5A7 by Directed Evolution for Good Performance of Three Properties. *Journal of Agricultural and Food Chemistry* 66(50)**,** 13228-13235. doi: 10.1021/acs.jafc.8b05662.

DeChellis, A., Nemmaru, B., Sammond, D., Douglass, J., Patil, N., Reste, O., et al. (2024). Supercharging Carbohydrate Binding Module Alone Enhances Endocellulase Thermostability, Binding, and Activity on Cellulosic Biomass. *ACS Sustainable Chemistry & Engineering* 12(9)**,** 3500-3516. doi: 10.1021/acssuschemeng.3c06266.

Fei, L., Jingcong, X., Xuesong, Z., and Linguo, Z. (2015). Improvement of the Optimum pH of *Aspergillus niger* Xylanase towards an Alkaline pH by Site-Directed Mutagenesis. *Journal of Microbiology and Biotechnology* 25(1)**,** 11-17. doi: 10.4014/jmb.1402.02055.

Gao, L., Guo, Q., Xu, R., Dong, H., Zhou, C., Yu, Z., et al. (2023). Loop optimization of *Trichoderma reesei* endoglucanases for balancing the activity–stability trade-off through cross-strategy between machine learning and the B-factor analysis. *Global Change Biology Bioenergy* 15(2)**,** 128-142. doi: 10.1111/gcbb.13011.

Goedegebuur, F., Dankmeyer, L., Gualfetti, P., Karkehabadi, S., Hansson, H., Jana, S., et al. (2017). Improving the thermal stability of cellobiohydrolase Cel7A from *Hypocrea jecorina* by directed evolution. *Journal of Biological Chemistry* 292(42)**,** 17418-17430. doi: 10.1074/jbc.M117.803270.

Han, C., Liu, Y., Liu, M., Wang, S., and Wang, Q. (2020). Improving the thermostability of a thermostable endoglucanase from *Chaetomium thermophilum* by engineering the conserved noncatalytic residue and N-glycosylation site. *International Journal of Biological Macromolecules* 164**,** 3361-3368. doi: 10.1016/j.ijbiomac.2020.08.225.

Han, N., Miao, H., Ding, J., Li, J., Mu, Y., Zhou, J., et al. (2017). Improving the thermostability of a fungal GH11 xylanase via site-directed mutagenesis guided by sequence and structural analysis. *Biotechnology for Biofuels* 10**,** 133. doi: 10.1186/s13068-017-0824-y.

Hegazy, U.M., El-Khonezy, M.I., Shokeer, A., Abdel-Ghany, S.S., Bassuny, R.I., Barakat, A.Z., et al. (2019). Revealing of a novel xylose-binding site of *Geobacillus stearothermophilus* xylanase by directed evolution. *The Journal of Biochemistry* 165(2)**,** 177-184. doi: 10.1093/jb/mvy092.

Huang, Q., Wang, K., Li, H., Yi, S., and Zhao, X. (2020). Enhancing cellulosic ethanol production through coevolution of multiple enzymatic characteristics of β-glucosidase from *Penicillium oxalicum* 16. *Applied Microbiology and Biotechnology* 104(19)**,** 8299-8308. doi: 10.1007/s00253-020-10858-8.

Kamale, C., Rauniyar, A., and Bhaumik, P. (2025). Rational design facilitates the improvement of glucose tolerance and catalytic properties of a beta-glucosidase from *Acetivibrio thermocellus*. *The FEBS Journal* 292(5)**,** 1174-1196. doi: 10.1111/febs.17394.

Khalili Ghadikolaei, K., Akbari Noghabi, K., and Shahbani Zahiri, H. (2017). Development of a bifunctional xylanase-cellulase chimera with enhanced activity on rice and barley straws using a modular xylanase and an endoglucanase procured from camel rumen metagenome. *Applied Microbiology and Biotechnology* 101(18)**,** 6929-6939. doi: 10.1007/s00253-017-8430-2.

Lai, Z., Zhou, C., Ma, X., Xue, Y., and Ma, Y. (2020). Enzymatic Characterization of a Novel Thermostable and Alkaline Tolerant GH10 Xylanase and Activity Improvement by Multiple Rational Mutagenesis Strategies. *International Journal of Biological Macromolecules* 170**,** 164-177. doi: 10.1016/j.ijbiomac.2020.12.137.

Li, Q., Sun, B., Jia, H., Hou, J., Yang, R., Xiong, K., et al. (2017a). Engineering a Xylanase from *Streptomyce Rochei* L10904 by Mutation to Improve Its Catalytic Characteristics. *International Journal of Biological Macromolecules* 101. doi: 10.1016/j.ijbiomac.2017.03.135.

Li, T., Wang, R., Hua, B., Cao, L., Zhang, Q., Zhai, Y., et al. (2025). Improving the Thermal Stability of GH11 Xylanase XynASP through Cord Region Engineering. *Journal of Agricultural and Food Chemistry* 73(2)**,** 1516-1528. doi: 10.1021/acs.jafc.4c10256.

Li, X.Q., Xia, J.L., Zhu, X.Y., Bilal, M., Tan, Z.B., and Shi, H. (2019). Construction and characterization of bifunctional cellulases: Caldicellulosiruptor-sourced endoglucanase, CBM, and exoglucanase for efficient degradation of lignocellulose. *Biochemical Engineering Journal* 151. doi: 10.1016/j.bej.2019.107363.

Li, Y., Zhang, H., Fu, Y., Zhou, Z., Yu, W., Zhou, J., et al. (2024). Enhancing Acid Resistance of *Aspergillus niger* Pectin Lyase through Surface Charge Design for Improved Application in Juice Clarification. *Journal of Agricultural and Food Chemistry* 72(20)**,** 11652-11662. doi: 10.1021/acs.jafc.4c01505.

Li, Y.X., Yi, P., Yan, Q.J., Qin, Z., Liu, X.Q., and Jiang, Z.Q. (2017b). Directed evolution of a β-mannanase from *Rhizomucor miehei* to improve catalytic activity in acidic and thermophilic conditions. *Biotechnology for Biofuels* 10(1)**,** 143. doi: 10.1186/s13068-017-0833-x.

Liang, Q., Zhan, Y., Yuan, M., Cao, L., Zhu, C., Mou, H., et al. (2021). Improvement of the Catalytic Ability of a Thermostable and Acidophilic beta-Mannanase Using a Consensus Sequence Design Strategy. *Frontiers in Microbiology* 12**,** 722347. doi: 10.3389/fmicb.2021.722347.

Liang, S., Lin, X., He, L., Liu, Y., Xiong, W., Lin, M., et al. (2025). Development of an efficient chimeric trifunctional xylanase/glucanase/feruloyl esterase and its application in the bioconversion of corn stover into fermentable sugars. *International Journal of Biological Macromolecules* 307(Pt 4)**,** 142236. doi: 10.1016/j.ijbiomac.2025.142236.

Lu, M., Li, Z., Zhuang, H., Yang, S., Zhao, X., Feng, R., et al. (2024). Dual Enhancement of Thermostability and Activity of Xylanase through Computer-Aided Rational Design. *ACS Sustainable Chem Eng* 12(41)**,** 15114-15124. doi: 10.1021/acssuschemeng.4c04974.

Lv, K., Li, X., Chen, K., Wu, B., He, B., and Schenk, G. (2024). Improving the Catalytic Efficiency of a GH5 Processive Endoglucanase by a Combinatorial Strategy Using Consensus Mutagenesis and Loop Engineering. *ACS Catalysis* 14(9)**,** 6856-6867. doi: 10.1021/acscatal.4c01083.

Lv, K., Shao, W., Pedroso, M.M., Peng, J., Wu, B., Li, J., et al. (2021). Enhancing the catalytic activity of a GH5 processive endoglucanase from *Bacillus subtilis* BS-5 by site-directed mutagenesis. *International Journal of Biological Macromolecules* 168**,** 442-452. doi: 10.1016/j.ijbiomac.2020.12.060.

Pavic, Q., Pillot, A., Tasseau, O., Legentil, L., and Tranchimand, S. (2019). Improvement of the versatility of an arabinofuranosidase against galactofuranose for the synthesis of galactofuranoconjugates. *Organic & Biomolecular Chemistry* 17(28)**,** 6799-6808. doi: 10.1039/c9ob01162e.

Qi, L., Zhang, S., Cao, F., Li, Y., Ke, Z., Wang, M., et al. (2025). Surface Charge Engineering Unlocks Dual Enhancement of Thermostability and Catalytic Efficiency in a Bacterial β‑Glucosidase for Sustainable Resveratrol Production. *Journal of Agricultural and Food Chemistry* 73(41)**,** 26303-26312. doi: 10.1021/acs.jafc.5c08213.

Ribeiro, L.F., Tullman, J., Nicholes, N., Silva, S.R., Vieira, D.S., Ostermeier, M., et al. (2016). A xylose-stimulated xylanase-xylose binding protein chimera created by random nonhomologous recombination. *Biotechnology for Biofuels* 9**,** 119. doi: 10.1186/s13068-016-0529-7.

Safran, J., Ung, V., Bouckaert, J., Habrylo, O., Molinie, R., Fontaine, J.X., et al. (2023). The specificity of pectate lyase *Vd*PelB from *Verticilium dahliae* is highlighted by structural, dynamical and biochemical characterizations. *International Journal of Biological Macromolecules* 231**,** 123137. doi: 10.1016/j.ijbiomac.2023.123137.

Santos, C.A., Morais, M.A.B., Terrett, O.M., Lyczakowski, J.J., Zanphorlin, L.M., Ferreira-Filho, J.A., et al. (2019). An engineered GH1 β-glucosidase displays enhanced glucose tolerance and increased sugar release from lignocellulosic materials. *Scientific Reports* 9(1)**,** 4903. doi: 10.1038/s41598-019-41300-3.

Santos, C.A., Zanphorlin, L.M., Crucello, A., Tonoli, C.C.C., Ruller, R., Horta, M.A.C., et al. (2016). Crystal Structure and Biochemical Characterization of the Recombinant ThBgl, a GH1 Β-Glucosidase Overexpressed in *Trichoderma Harzianum* under Biomass Degradation Conditions. *Biotechnology for biofuels* 9(1). doi: 10.1186/s13068-016-0487-0.

Sun, D., Li, C., Cui, P., Zhang, J., Zhou, Y., Wu, M., et al. (2022). Reshaping the binding channel of a novel GH113 family β-mannanase from *Paenibacillus cineris* (*Pc*Man113) for enhanced activity. *Bioresources and Bioprocessing* 9(1)**,** 17. doi: 10.1186/s40643-022-00505-7.

Tan, S., Tao, X., Zheng, P., Chen, P., Yu, X., Li, N., et al. (2023). Thermostability modification of β-mannanase from *Aspergillus niger* via flexibility modification engineering. *Frontiers in Microbiology* 14**,** 1119232. doi: 10.3389/fmicb.2023.1119232.

Tu, T., Li, Y., Su, X., Meng, K., Ma, R., Wang, Y., et al. (2016). Probing the role of cation-pi interaction in the thermotolerance and catalytic performance of endo-polygalacturonases. *Scientific Reports* 6**,** 38413. doi: 10.1038/srep38413.

Wang, H., Qi, X., Gao, S., Kan, G., Damdindorj, L., An, Y., et al. (2024). Characterization of a novel multifunctional β-glucosidase/xylanase/feruloyl esterase and its effects on improving the quality of Longjing tea. *Food Chemistry* 453**,** 139637. doi: 10.1016/j.foodchem.2024.139637.

Wang, H., Qi, X., Gao, S., Zhang, Y., and An, Y. (2022). Biochemical characterization of an engineered bifunctional xylanase/feruloyl esterase and its synergistic effects with cellulase on lignocellulose hydrolysis. *Bioresource Technology* 355**,** 127244. doi: 10.1016/j.biortech.2022.127244.

Wang, L., Cao, K., Pedroso, M.M., Wu, B., Gao, Z., He, B., et al. (2021). Sequence- and structure-guided improvement of the catalytic performance of a GH11 family xylanase from *Bacillus subtilis*. *Journal of Biological Chemistry* 297(5)**,** 101262. doi: 10.1016/j.jbc.2021.101262.

Wang, W., Andric, N., Sarch, C., Silva, B.T., Tenkanen, M., and Master, E.R. (2018a). Constructing arabinofuranosidases for dual arabinoxylan debranching activity. *Biotechnology and Bioengineering‌* 115(1)**,** 41-49. doi: 10.1002/bit.26445.

Wang, X.-C., You, S.-P., Zhang, J.-X., Dai, Y.-M., Zhang, C.-Y., Qi, W., et al. (2018b). Rational design of a thermophilic β-mannanase from *Bacillus subtilis* TJ-102 to improve its thermostability. *Enzyme Microb Technol* 118**,** 50-56. doi: 10.1016/j.enzmictec.2018.07.005.

Wang, X., Lu, Z., Xu, T., Selvaraj, J.N., Yi, L., and Zhang, G. (2018c). Improving the specific activity and thermo-stability of alkaline pectate lyase from *Bacillus subtilis* 168 for bioscouring. *Biochemical Engineering Journal* 129**,** 74-83. doi: 10.1016/j.bej.2017.11.001.

Whitehead, T.A., Bandi, C.K., Berger, M., Park, J., and Chundawat, S.P.S. (2017). Negatively Supercharging Cellulases Render Them Lignin-Resistant. *ACS Sustainable Chemistry & Engineering* 5(7)**,** 6247-6252. doi: 10.1021/acssuschemeng.7b01202.

Wu, Q., Zhang, C., Dong, W., Lu, H., Yang, Y., Li, W., et al. (2023a). Simultaneously Enhanced Thermostability and Catalytic Activity of Xylanase from *Streptomyces rameus* L2001 by Rigidifying Flexible Regions in Loop Regions of the N-Terminus. *Journal of Agricultural and Food Chemistry* 71(34)**,** 12785-12796. doi: 10.1021/acs.jafc.3c03871.

Wu, Q., Zhang, C., Zhu, W., Lu, H., Li, X., Yang, Y., et al. (2023b). Improved thermostability, acid tolerance as well as catalytic efficiency of *Streptomyces rameus* L2001 GH11 xylanase by N-terminal replacement. *Enzyme and Microbial Technology* 162**,** 110143. doi: 10.1016/j.enzmictec.2022.110143.

Xia, W., Xu, X., Qian, L., Shi, P., Bai, Y., Luo, H., et al. (2016). Engineering a highly active thermophilic β-glucosidase to enhance its pH stability and saccharification performance. *Biotechnology for Biofuels* 9**,** 147. doi: 10.1186/s13068-016-0560-8.

Xiang, L., Lu, Y.H., Wang, H., Wang, M.X., and Zhang, G.M. (2019). Improving the specific activity and pH stability of xylanase XynHBN188A by directed evolution. *Bioresources and Bioprocessing* 6. doi: 10.1186/s40643-019-0262-8.

Xue, J., Jiang, X., Li, A., Li, J., Su, X., Huang, J., et al. (2025). Catalytic Efficiency Improvement in Cellobiohydrolase I by Cross-Species Domain Exchange Engineering. *International Journal of Molecular Sciences* 26(9). doi: 10.3390/ijms26094024.

Zhang, W., Liu, Z., Zhou, S., Mou, H., and Zhang, R. (2019). Cloning and Expression of a Β-Mannanase Gene from *Bacillus* sp. MK-2 and Its Directed Evolution by Random Mutagenesis. *Enzyme and Microbial Technology‌* 124**,** 70-78. doi: 10.1016/j.enzmictec.2019.02.003.

Zhang, Z., Li, Z., Yang, M., Zhao, F., and Han, S. (2024). Machine learning-guided multi-site combinatorial mutagenesis enhances the thermostability of pectin lyase. *International Journal of Biological Macromolecules* 277**,** 134530. doi: 10.1016/j.ijbiomac.2024.134530.

Zhang, Z., Zhang, Z., Yu, Z., Chen, S., Zhang, M., Zhang, T., et al. (2022). Simultaneous Improvement of Final Product-Tolerance and Thermostability of GH39 Xylosidase for Prebiotic Production by Directed Evolution. *Foods* 11(19). doi: 10.3390/foods11193039.

Zheng, F., Vermaas, J.V., Zheng, J., Wang, Y., Tu, T., Wang, X., et al. (2019). Activity and Thermostability of GH5 Endoglucanase Chimeras from Mesophilic and Thermophilic Parents. *Applied and Environmental Microbiology* 85(5). doi: 10.1128/AEM.02079-18.

Zhou, C., Cao, Y., Xue, Y., Liu, W., Ju, J., and Ma, Y. 2023. Structure of an Alkaline Pectate Lyase and Rational Engineering with Improved Thermo-Alkaline Stability for Efficient Ramie Degumming. *International Journal of Molecular Sciences* [Online], 24(1).

Zhou, X., Xu, Z., Li, Y., He, J., and Zhu, H. (2022). Improvement of the Stability and Activity of an LPMO Through Rational Disulfide Bonds Design. *Frontiers in Bioengineering and Biotechnology* Volume 9 - 2021. doi: 10.3389/fbioe.2021.815990.

Zhou, Z., and Wang, X. (2021). Rational design and structure-based engineering of alkaline pectate lyase from *Paenibacillus* sp. 0602 to improve thermostability. *BMC Biotechnology* 21(1)**,** 32. doi: 10.1186/s12896-021-00693-8.
